# The SET-domain protein CgSet4 negatively regulates antifungal drug resistance *via* the ergosterol biosynthesis transcriptional regulator CgUpc2a

**DOI:** 10.1016/j.jbc.2022.102485

**Published:** 2022-09-13

**Authors:** Priyanka Bhakt, Mayur Raney, Rupinder Kaur

**Affiliations:** 1 Laboratory of Fungal Pathogenesis, Centre for DNA Fingerprinting and Diagnostics, Hyderabad, India; 2 Graduate Studies, Manipal Academy of Higher Education, Manipal, Karnataka, India

**Keywords:** multidrug transporter, drug resistance, fungi, cell wall, microbial pathogenesis, SET domain, fluconazole, caspofungin, ergosterol biosynthesis genes, Set4, CAA, casamino acid, CDFD, Centre for DNA Fingerprinting and Diagnostics, ChIP, chromatin immunoprecipitation, CSP, caspofungin, C_T_, cycle threshold, DAVID, Database for Annotation, Visualization, and Integrated Discovery, DEG, differentially expressed gene, DHE, dehydroergosterol, FA, fatty acid, FLC, fluconazole, MDR, multidrug resistance, qRT–PCR, quantitative RT–PCR, SET, {suppressor of variegation 3 to 9 [Su(var)3–9], enhancer of zeste [E(z)], and trithorax (Trx)}, SRE, sterol regulatory element, YPD, yeast extract–peptone–dextrose

## Abstract

Invasive fungal infections, which pose a serious threat to human health, are increasingly associated with a high mortality rate and elevated health care costs, owing to rising resistance to current antifungals and emergence of multidrug-resistant fungal species. *Candida glabrata* is the second to fourth common cause of *Candida* bloodstream infections. Its high propensity to acquire resistance toward two mainstream drugs, azoles (inhibit ergosterol biosynthesis) and echinocandins (target cell wall), in clinical settings, and its inherent low azole susceptibility render antifungal therapy unsuccessful in many cases. Here, we demonstrate a pivotal role for the SET {suppressor of variegation 3 to 9 [Su(var)3–9], enhancer of zeste [E(z)], and trithorax (Trx)} domain–containing protein, CgSet4, in azole and echinocandin resistance *via* negative regulation of multidrug transporter–encoding and ergosterol biosynthesis (*ERG*) genes through the master transcriptional factors CgPdr1 and CgUpc2A, respectively. RNA-Seq analysis revealed that *C. glabrata* responds to caspofungin (CSP; echinocandin antifungal) stress by downregulation and upregulation of *ERG* and cell wall organization genes, respectively. Although CgSet4 acts as a repressor of the ergosterol biosynthesis pathway *via CgUPC2A* transcriptional downregulation, the CSP-induced *ERG* gene repression is not dependent on CgSet4, as CgSet4 showed diminished abundance on the *CgUPC2A* promoter in CSP-treated cells. Furthermore, we show a role for the last three enzymes of the ergosterol biosynthesis pathway, CgErg3, CgErg5, and CgErg4, in antifungal susceptibility and virulence in *C. glabrata*. Altogether, our results unveil the link between ergosterol biosynthesis and echinocandin resistance and have implications for combination antifungal therapy.

Fungal infections, whose incidence has been on the rise over last 2 decades, are associated with a mortality rate of as high as 95% (1, 2, 3, 4, 5). Infections caused by the *Candida* species are the most prevalent cause of hospital-acquired bloodstream fungal infections, with *Candida albicans* being the predominant species (1, 2, 4, 5). *Candida glabrata* is emerging as the first to third most prevalent *non-albicans Candida* species, after *Candida tropicalis* and *Candida parapsilosis* (1, 2, 4, 5, 6).

*C. glabrata* is an asexual haploid budding yeast, which belongs to the *Nakaseomyces* clade (7, 8). It shares a common ancestor with the non-pathogenic yeast *Saccharomyces cerevisiae* and is distinct in its virulence traits from other prevalent pathogenic species of *Candida* (7, 8). *C. glabrata* lacks secreted proteolytic activity, does not form true hyphae, and displays elevated levels of stress resistance (7, 8). Based on its position in the phylogenetic tree and close genetic relatedness with *S. cerevisiae*, pathogenesis mechanisms in *C. glabrata* are postulated to have arisen independently of other *Candida* pathogens (7, 8). The ability to survive and replicate in human macrophages, suppress host innate immune response, form biofilms, adhere to host tissue, metabolic flexibility and high resistance to diverse stresses including antifungals, assist *C. glabrata* establish successful mucosal and invasive infections (1, 7, 8).

The incidence of *C. glabrata* infections has been on rise since last 2 decades, and alarmingly, this surge is also associated with an increased prevalence of co-resistance to two mainstream antifungal agents, azoles and echinocandins, thereby, hampering the success of antifungal therapy (3, 4, 5, 9, 10, 11, 12). Polyene, azole, and echinocandin drugs are largely being used in hospitals worldwide to treat bloodstream fungal infections (11, 12, 13). The target of azole antifungals, which are fungistatic, is an enzyme of the ergosterol biosynthesis pathway, lanosterol 14-alpha-demethylase enzyme, that is encoded by the *ERG11* gene (13, 14). Azole drug–induced growth inhibition is attributed to ergosterol depletion from the plasma membrane and accumulation of the toxic sterol intermediates (13, 14). Contrary to azoles, the echinocandin antifungals target the β-glucan synthase enzyme, which is encoded by the family of *FKS* genes, and is involved in the synthesis of the cell wall structural polymer 1,3-β-d-glucan (11, 12, 13, 15). The polyene class of antifungals bind to ergosterol and disrupt plasma membrane integrity as well as extract ergosterol from the cell membrane (12, 13).

*C. glabrata* is inherently less susceptible to azole drugs, and *C. glabrata* infections are increasingly being associated with azole and/or echinocandin resistance (4, 5, 9, 10, 11, 12). The elevated expression of ATP-binding cassette class of multidrug transporters is the primary mode of azole resistance in *C. glabrata* in hospitals worldwide (11, 12, 14). Genes coding for multidrug transporters are under the regulation of a Zn_2_Cys_6_ binuclear zinc cluster domain–containing transcription factor, which is encoded by the *CgPDR1* gene (14, 16). A large number of single amino acid substitution mutations in *CgPDR1* have been reported in azole-resistant clinical isolates of *C. glabrata* (14, 17, 18). CgCdr1 and CgCdr2 represent two major multidrug transporters in *C. glabrata*, whose elevated expression is associated with increased azole efflux, and the resultant decreased intracellular accumulation of azole drugs (14, 19, 20).

Another key regulator of azole antifungal response is the Zn_2_Cys_6_ transcription factor CgUpc2A (Cagl0c01199p), which is required for basal and induced expression of the ergosterol biosynthesis (*ERG*) genes, with several *ERG* genes showing significant upregulation upon azole exposure (21, 22, 23). Intriguingly, CgUpc2A has recently been shown to bind to *CgPDR1* and *CgCDR1* promoter sequences, thereby linking ergosterol biosynthesis with the CgPdr1-mediated transcriptional regulatory network that controls expression of multidrug resistance (MDR) genes (24).

*C. glabrata* possesses three *CgFKS* genes, and point mutations in the hot spot regions of *CgFKS1* and *CgFKS2* genes largely account for echinocandin resistance in clinical isolates of *C. glabrata* (15, 25). These hot spot regions represent echinocandin-binding regions, with mutations decreasing the binding affinity of echinocandins for the β-1,3-d-glucan synthase enzyme (12, 15). Besides elevated *CgFKS* gene transcription, the echinocandin exposure also leads to increased expression of the chitin synthase genes, thereby resulting in compensatory activation of chitin synthesis in the cell wall (15, 26). Mutations in the *CgMSH2* gene, that codes for a component of the DNA mismatch repair pathway, have been associated with MDR in some isolates of *C. glabrata* (11, 15).

We have recently reported that disruption of *CgSET2* (encodes a histone H3 lysine 36 methyltransferase), *CgSET4* (contains evolutionarily conserved SET {suppressor of variegation 3 to 9 [Su(var)3–9], enhancer of zeste [E(z)], and trithorax (Trx)} domain), and *CgRPH1* (encodes a putative histone lysine demethylase) genes resulted in azole resistance, resistance, and sensitivity, respectively (27, 28). Notably, these *C. glabrata* proteins have orthologs in *S. cerevisiae*, Set2, Set4, and Rph1, which have been studied for their role in chromatin homeostasis (29, 30, 31, 32). Rph1, a JmjC domain–containing histone lysine demethylase, and Set2, a methyltransferase, have been implicated in demethylation and methylation of the lysine 36 residue in histone H3, respectively, in *S. cerevisiae* (29, 32). Set4 in *S. cerevisiae* has been shown to be a stress-responsive chromatin-associated protein that regulates gene expression under oxidative stress conditions, acts as a repressor of ergosterol biosynthesis, and aids in the maintenance of a repressive environment at subtelomeres (31, 33, 34). However, the epigenetic regulation of MDR genes is largely unstudied in *C. glabrata*.

Here, we report that of six SET domain proteins (CgSet1–CgSet6) in *C. glabrata*, CgSet4 uniquely acts as a repressor of CgPdr1-dependent MDR and CgUpc2a-dependent ergosterol biosynthesis pathways. Besides showing that *CgSET4* deletion results in decreased susceptibility to fluconazole (FLC) and caspofungin (CSP) drugs, we also report that ergosterol biosynthesis is downregulated in response to CSP stress. We further show that CgSet4-dependent negative regulation of *CgPDR1* and *CgERG* genes is mediated through CgUpc2a. Finally, our animal infection studies reveal a role for the master transcriptional factor CgUpc2a, and two enzymes (CgErg3 and CgErg4) of the ergosterol biosynthesis pathway, as well as, for all CgSet proteins but for CgSet6, in survival of *C. glabrata* in the mouse systemic candidiasis model. Besides uncovering a link between ergosterol biosynthesis and echinocandin resistance, our findings yield key insights into the intertwined transcriptional networks that regulate cellular response to two seemingly distinct stresses, cell wall impairment, and ergosterol synthesis inhibition.

## Results

### The SET domain–containing protein CgSet4 negatively regulates azole and CSP resistance

**Figure S1:**
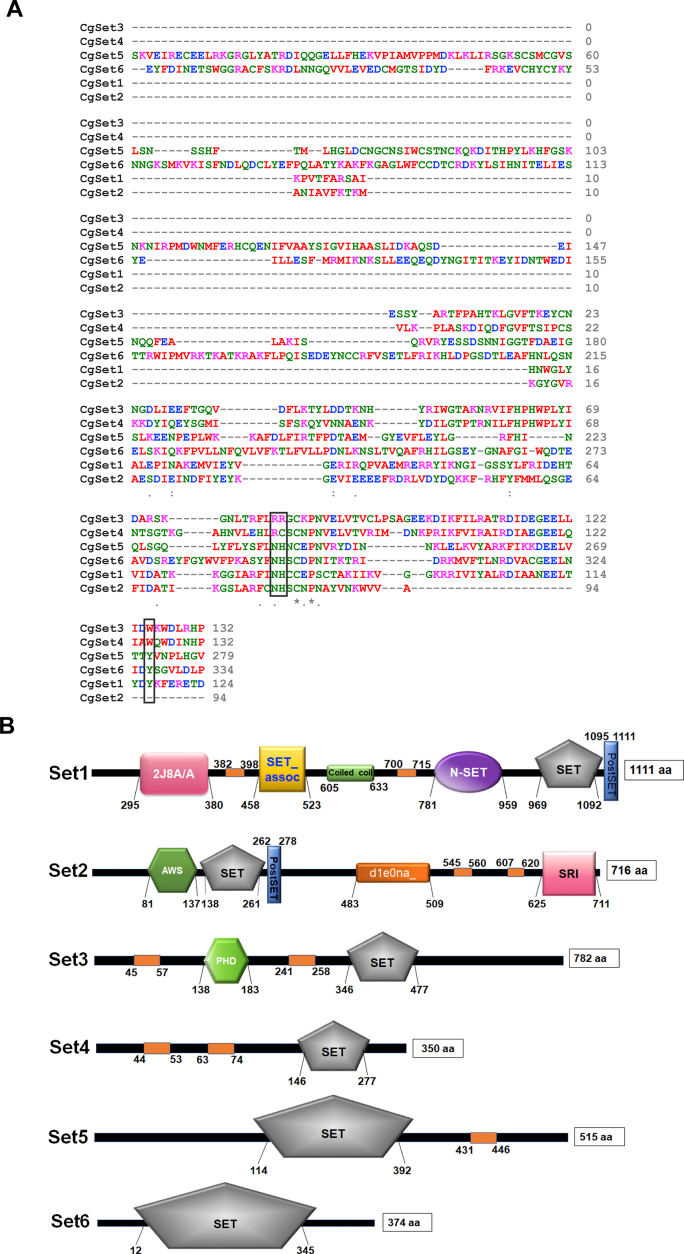
***C. glabrata*contains six SET domain-containing proteins.**

We have previously shown a role for putative histone chaperones CgFpr3 and CgFpr4, histone demethylase CgRph1, and histone H3K36-specific methyltransferase CgSet2 in regulating *CgPDR1*-dependent expression of multidrug transporter–encoding genes, and/or resistance to azole antifungals (28). Extending our results further, we, here, have examined the contribution of *C. glabrata* proteins, which possess the evolutionarily conserved SET domain, to resistance toward azole and echinocandin drugs. The SET domain consists of about 130 conserved amino acids (35). Several SET domain–containing proteins are known to methylate both histone and nonhistone proteins, with ε-amino group of the lysine residue in histones being able to undergo monomethylation, dimethylation, and trimethylation (35, 36, 37). Through *in silico* analysis, we first identified six genes, *CgSET1–SET6*, in the *C. glabrata* genome, which code for proteins containing the SET domain. The key features of these six SET domain–containing proteins CgSet1–CgSet6 and functions of their *S. cerevisiae* orthologs are listed in Table S1. The amino acid sequence alignment of the SET domain, and the predicted domain organization of CgSet1–Set6 proteins are shown in Fig. S1, *A* and *B*, respectively. Notably, CgSet1 and CgSet2 enzymes have recently been shown to be required for monomethylation, dimethylation, and trimethlyation of lysine-4, and trimethylation of lysine-36 residues, in histone H3, respectively (28, 38), while this work was underway.

**Figure 1:**
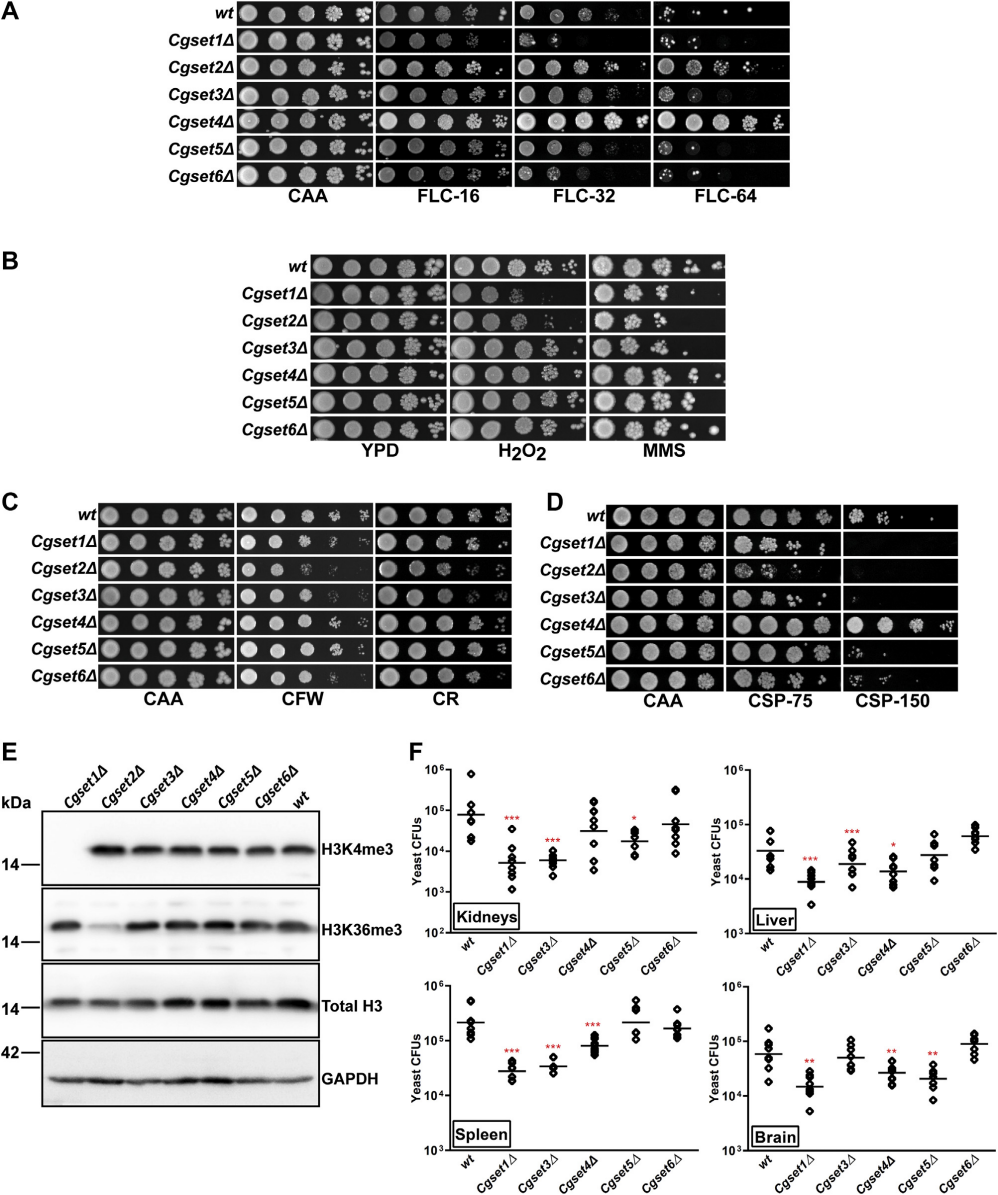
**The loss of SET domain–containing protein, CgSet4, leads to azole and echinocandin resistance.***A*, serial dilution spotting analysis illustrating fluconazole (FLC) susceptibility of indicated *Candida glabrata* strains. Overnight-grown *C. glabrata* cultures were 10-fold serially diluted, and 3 μl of each dilution was spotted on the CAA medium lacking (CAA) or containing 16 μg/ml FLC (FLC-16), 32 μg/ml FLC (FLC-32), or 64 μg/ml FLC (FLC-64). Images were captured after 2 days of growth at 30 °C. “*wt*” denotes the *wild-type* (*wt)* strain. *B*, serial dilution cell spotting analysis illustrating oxidative stress and DNA damage stress susceptibility of indicated *C. glabrata* strains. Hydrogen peroxide (H_2_O_2_) and methyl methane sulfonate (MMS) were used at a concentration of 35 mM and 0.04%, respectively, in YPD medium. *C*, serial dilution cell spotting analysis illustrating cell wall stress susceptibility of indicated *C. glabrata* strains. Both calcofluor white (CFW) and Congo Red were used at a concentration of 2 mg/ml in CAA medium. *D*, liquid medium–based growth analysis illustrating caspofungin (CSP) susceptibility of indicated *C. glabrata* strains. *C. glabrata* strains were cultured at 30 ^ο^C in CAA medium lacking (CAA) or containing 75 ng/ml CSP (CSP-75) or 150 ng/ml CSP (CSP-150) for 16 h. After incubation, cultures were diluted in PBS, and 3 μl of 5-, 25-, 125-, and 625-fold diluted cultures were spotted on CAA medium, and growth was recorded after 1 day of growth at 30 °C. *E*, representative immunoblots showing trimethylation on 4th and 36th lysine residues in histone H3 in indicated *C. glabrata* strains. Log-phase *wt* and *CgsetΔ* cells were collected, washed with PBS, and lysed with glass beads. Whole-cell lysates (50 μg protein) were resolved on 15% SDS-PAGE and probed with anti-H3, anti-H3K4me3, anti-H3K36me3, and anti-GAPDH antibodies. Bands representing histone H3 and CgGapdh proteins corresponded to about 17 and 36 kDa, respectively. CgGapdh was used as a loading control. *F*, organ fungal load in 6- to 8-week-old female BALB/c mice was determined 7 days postintravenous infection with indicated *C. glabrata* strains (4 × 10^7^ cells). *Diamonds* and *bars* denote CFUs recovered from target organs of the individual mouse, and the CFU geometric mean (n = 7–10), respectively, for each organ. ∗*p* < 0.05; ∗∗*p* < 0.01; ∗∗∗*p* < 0.001; Mann–Whitney test. CAA, casamino acid; CFU, colony-forming unit; SET, {suppressor of variegation 3 to 9 [Su(var)3–9], enhancer of zeste [E(z)], and trithorax (Trx)}; YPD, yeast extract–peptone–dextrose.

Next, we generated and characterized deletion strains for *CgSET1*, and *CgSET3–6* genes, as we had recently shown that the loss of *CgSET2* gene resulted in decreased sensitivity to the azole drug FLC (28). In consistence with the recent studies (28, 38), phenotypic analysis showed increased sensitivity and a moderate level of resistance toward FLC in *Cgset1Δ* and *Cgset2Δ* mutants, respectively, compared with *wild-type* (*wt*) cells (Fig. 1*A*). Contrarily, the *Cgset4Δ* mutant exhibited a high level of FLC resistance (Fig. 1*A*). In addition, an increased susceptibility of *Cgset1Δ* and *Cgset2Δ* mutants toward hydrogen peroxide (oxidative stressor) and elevated susceptibility of *Cgset1Δ*, *Cgset2Δ*, and *Cgset3Δ* mutants toward methyl methanesulfonate (DNA-damaging agent) were observed (Fig. 1*B*). Interestingly, *Cgset1Δ*, *Cgset2Δ*, *Cgset3Δ*, and *Cgset6Δ* mutants also displayed mild sensitivity to calcofluor white and congo red (cell wall stressors), compared with *wt* cells (Fig. 1*C*). No other notable phenotype was found for mutants lacking CgSet1–6 proteins. Besides implicating CgSet1–3 and CgSet6 proteins in survival of different stresses, these results indicate that CgSet1, and CgSet2 and CgSet4, modulate azole resistance albeit *via* opposite mode.

Furthermore, since we had previously reported that Tn*7* insertion in the *CgSET4* gene is associated with resistance to both FLC and CSP (echinocandin drug) (27), we next checked the role of SET domain–containing proteins in CSP tolerance in *C. glabrata*. We found the *CgSET4* gene loss to be uniquely associated with CSP resistance, whereas *Cgset1Δ*, *Cgset2Δ*, *Cgset3Δ*, and *Cgset6Δ* mutants exhibited varied levels of increased susceptibility to CSP, compared with *wt* cells (Fig. 1*D*). The *Cgset5Δ* mutant exhibited *wt*-like growth in the presence of CSP (Fig. 1*D*). Besides showing differential requirement of CgSet1–CgSet6 proteins in antifungal tolerance, these data collectively implicate CgSet4 in resistance to both cell membrane– (azole) and cell wall–targeting (echinocandin) antifungal drugs.

To ascribe histone H3 lysine methyltransferase activity to SET domain–containing proteins in *C. glabrata*, we performed Western analysis and examined trimethylation of lysine 4 and lysine 36 residues in histone H3 in *Cgset1Δ*–*Cgset6Δ* mutants. As reported recently (28, 38), the *Cgset1Δ* mutant exhibited no H3K4me3, whereas the *Cgset2Δ* mutant displayed very low levels of H3K36me3 (Fig. 1*E*). Notably, *CgSET3–SET6* gene loss neither had any effect on trimethylation of the H3K4 residue nor on the H3K36 residue (Fig. 1*E*). Our attempts to determine methylation at other lysine residues in H3 were not successful because of nonavailability and/or nonspecificity of commercially available antibodies. However, these data for CgSet3–CgSet6 proteins are in agreement with known functions of their counterparts in *S. cerevisiae*, as ScSet3, ScSet4, and ScSet6 proteins have not yet been associated with histone lysine methyltransferase activity, whereas ScSet5 has been shown to monomethylate lysines 5, 8, and 12 in histone H4 (30, 31, 39, 40).

Since we had recently reported a requirement for CgSet2 for survival in mice (28), we next assessed the survival of *Cgset1Δ* and *Cgset3Δ*–*Cgset6Δ* mutants in the murine model of systemic candidiasis. We found CgSet1 and CgSet3 to be required for survival of *C. glabrata* in kidneys, liver, and spleen, as 5- to 20-fold lower yeast colony-forming units were recovered from organs of the mice infected with *Cgset1Δ* and *Cgset3Δ* mutants, compared with the *wt*-infected mice (Fig. 1*F*). Contrarily, the *Cgset4Δ*-infected mice displayed about three-fold lower fungal burden in liver, spleen, and brain, as compared with the *wt*-infected mice (Fig. 1*F*). Of note, the *Cgset1Δ*-infected mice exhibited four-fold lower fungal burden in brain, whereas the *Cgset5Δ*-infected mice had eight-fold and three-fold lower fungal burden in kidneys and brain, respectively, of infected mice, as compared with the *wt*-infected mice (Fig. 1*F*). These data suggest that CgSet6 is not required for survival in the mouse systemic candidiasis model, whereas CgSet1, CgSet3, CgSet4, and CgSet5 are required in an organ-dependent manner, thereby underscoring the role of SET domain–containing proteins in virulence of *C. glabrata*.

### *CgSET4* expression is downregulated in response to antifungal exposure

**Figure 2:**
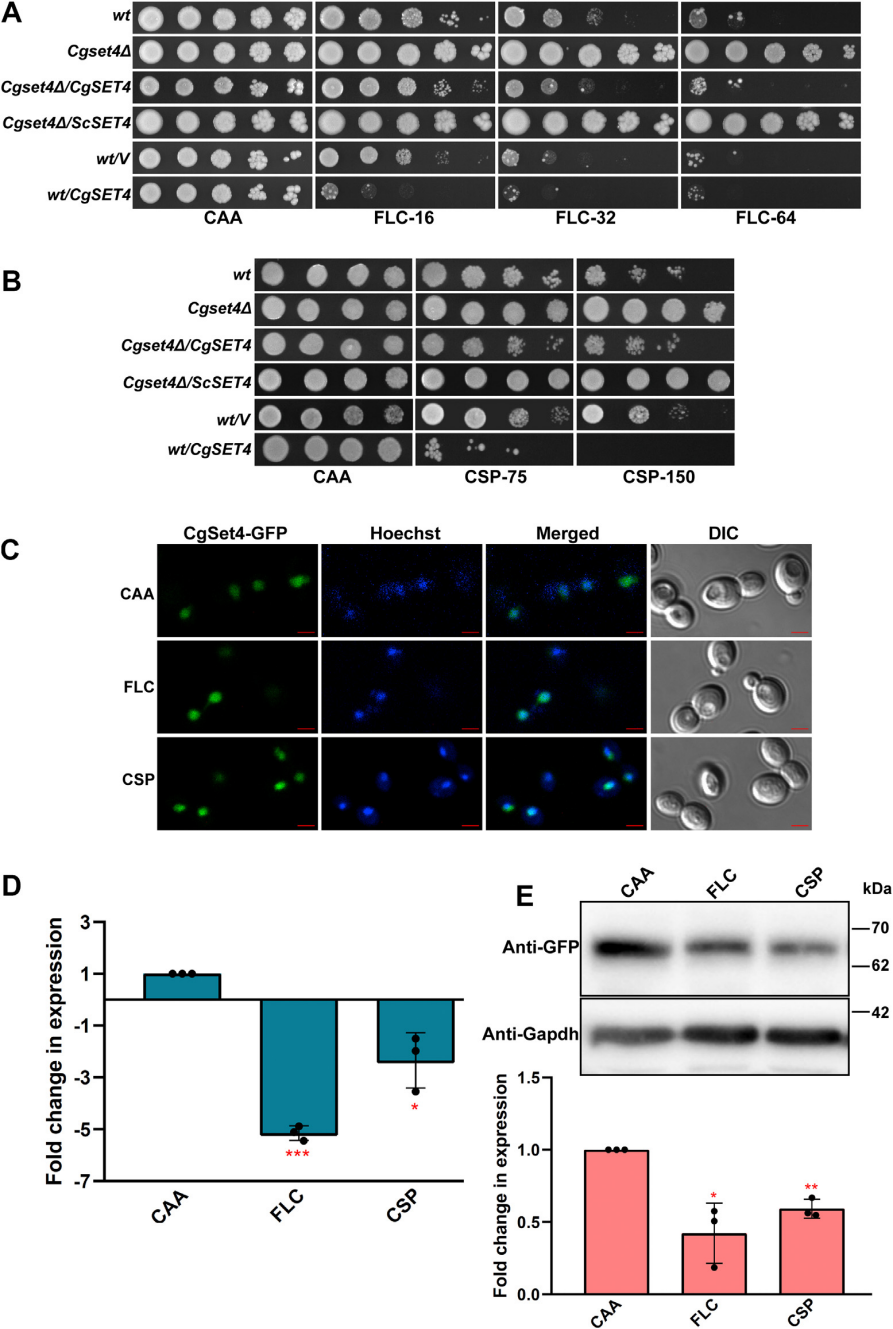
**Antifungal exposure results in diminished transcript and protein levels of the *CgSET4* gene.***A*, serial dilution spotting analysis illustrating fluconazole (FLC) susceptibility of indicated *Candida glabrata* strains. FLC was used at a concentration of 16 μg/ml (FLC-16), 32 μg/ml (FLC-32), and 64 μg/ml (FLC-64) in CAA medium. “*V*,” *CgSET4*, and *ScSET4* denote empty vector, *C. glabrata SET4*, and *Saccharomyces cerevisiae SET4* gene, respectively. *B*, liquid medium–based growth analysis illustrating caspofungin (CSP) susceptibility of indicated *C. glabrata* strains. CSP was used at a concentration of 75 ng/ml (CSP-75) and 150 ng/ml (CSP-150) in CAA medium. *C*, representative confocal microscopy images of CAA medium–grown logarithmic-phase *wt* cells expressing CgSet4-GFP ectopically showing colocalization of CgSet4-GFP with the Hoechst 33258–stained nuclei. *C. glabrata* strains were grown in CAA medium lacking (CAA) or containing 64 μg/ml FLC or 150 ng/ml CSP for 1 h and stained with 1 μg/ml Hoechst 33258 for 15 min at 37 ^ο^C. Cells were washed, suspended in PBS, and visualized using the Confocal microscope (Zeiss LSM 700 equipped with 63×/1.44 numerical aperture objective). DIC, differential interference contrast; Hoechst 33258, DNA-binding stain. Bar represents 2.0 μm. *D*, qRT–PCR analysis showing transcriptional downregulation of the *CgSET4* gene in response to FLC and CSP exposure. Log-phase *wt* cultures were left untreated or treated either with 64 μg/ml FLC or 250 ng/ml CSP for 1 h. RNA was isolated using the acid-phenol extraction method, and *CgSET4* transcript levels were measured by qRT–PCR using the 2^−ΔΔCt^ method. Data (mean ± SEM, n = 3) were normalized against the *CgTDH3* mRNA as control and represent fold change in *CgSET4* gene expression in FLC- or CSP-treated *wt* cells, as compared with the CAA medium–grown *wt* cells (taken as 1.0). ∗*p* < 0.05; ∗∗∗*p* < 0.001, paired two-tailed Student's *t* test. *E*, representative immunoblots showing reduced CgSet4-GFP levels upon FLC and CSP exposure. Log-phase *wt* cultures were left untreated or treated either with 64 μg/ml FLC or 250 ng/ml CSP for 1 h. Cultures were spun down, cells were collected, and whole-cell extracts were prepared by glass bead lysis method. Samples containing 60 μg protein were resolved on 12% SDS-PAGE and probed with anti-GFP and anti-GAPDH antibodies. CgGapdh was used as a loading control. The intensity of individual bands in three independent Western blots was quantified using the ImageJ densitometry software, and the CgSet4-GFP signal was normalized to the corresponding CgGapdh signal. Data (mean ± SEM, n = 3) represent fold change in CgSet4-GFP levels in FLC or CSP-treated *wt* cells, compared with CAA medium–grown *wt* cells (considered as 1.0) and are plotted as a bar graph underneath the blot images. ∗*p* < 0.05; ∗∗*p* < 0.01, paired two-tailed Student's *t* test. CAA, casamino acid; qRT–PCR, quantitative RT–PCR.

The high level of FLC and CSP resistance in the *Cgset4Δ* mutant prompted us to characterize this mutant further. Therefore, we first verified, through mutant complementation analysis, that the FLC and CSP resistance in the mutant is due to the lack of *CgSET4* gene. For this, we first cloned *CgSET4* under its own promoter and expressed it ectopically in the *Cgset4Δ* mutant. Next, we performed serial dilution spotting assay and found the reversal of FLC (Fig. 2*A*) and CSP (Fig. 2*B*) resistance in the *Cgset4Δ* mutant, as the *Cgset4Δ/CgSET4* strain displayed *wt*-like susceptibility to both antifungals (Fig. 2, *A* and *B*). Consistent with the *Cgset4Δ* mutant phenotype, overexpression of the *CgSET4* gene led to elevated sensitivity to FLC (Fig. 2*A*) and CSP (Fig. 2*B*), suggesting that CgSet4 negatively regulates resistance toward FLC and CSP drugs in *C. glabrata*.

CgSet4 is an uncharacterized 350 amino acid protein that showed 40% identity with its *S. cerevisiae* ortholog and contains a 132 amino acid (146–277 amino acids) SET domain (Fig. S1*B*). In *S. cerevisiae*, Set4 has a paralog, Set3; however, neither Set3 nor Set4 has been reported to possess lysine methyltransferase activity (31, 39). Similarly, we found normal H3K4me3 and H3K36me3 levels in the *Cgset4Δ* mutant (Fig. 1*E*). The amino acid sequence similarity and the lack of histone H3K4 and H3K36 methyltransferase activity in both CgSet4 and ScSet4 proteins prompted us to examine if *ScSET4* expression could reverse antifungal resistance in the *Cgset4Δ* mutant. For this, we ectopically expressed the *S. cerevisiae SET4* from the endogenous *CgSET4* promoter in the *Cgset4Δ* mutant and compared growth profiles of the *Cgset4Δ* mutant expressing either *ScSET4* or *CgSET4*, in the presence of antifungals. We found that unlike *CgSET4*, ectopic expression of the *S. cerevisiae SET4* could not rescue FLC and CSP resistance phenotype of the *Cgset4Δ* mutant (Fig. 2, *A* and *B*), indicating functional differences between CgSet4 and ScSet4 proteins in modulation of antifungal resistance. Of note, while the *SET4* gene loss led to azole resistance, it had no effect on CSP susceptibility in *S. cerevisiae* (33).

**Figure S2:**
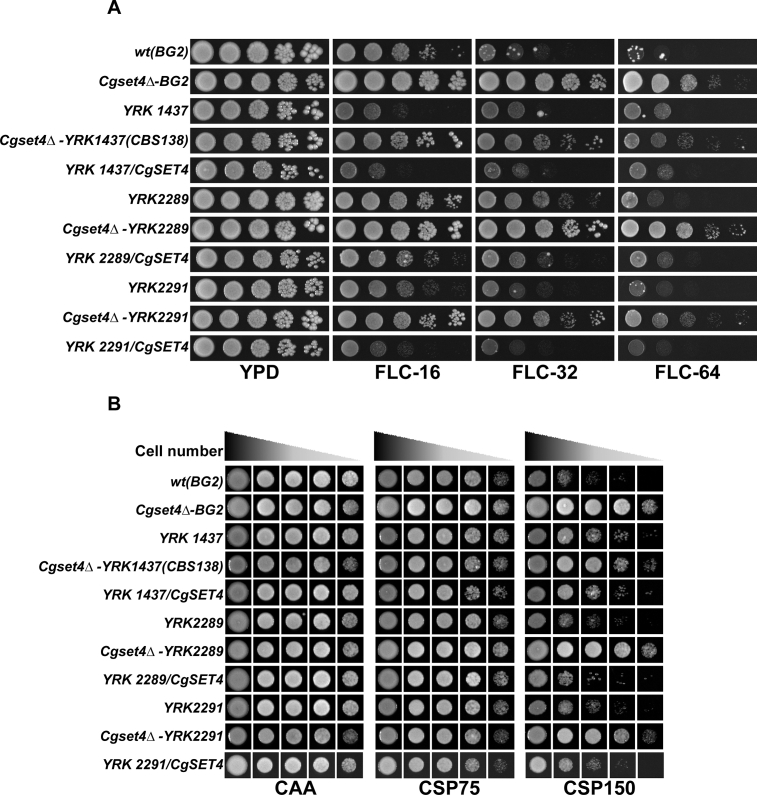
**CgSet4 negativelyregulates fluconazole and caspofungin resistance in clinical strains of *C. glabrata.***

To examine the role of CgSet4 in other clinical strains of *C. glabrata*, we deleted *CgSET4* gene in the reference strain CBS138 and two Indian clinical isolates YRK2289 and YRK2291 (27). The *CgSET4* loss resulted in decreased susceptibility to FLC (Fig. S2*A*) and CSP (Fig. S2*B*) in all three strain backgrounds. Furthermore, *CgSET4* overexpression led to FLC (Fig. S2*A*) and CSP (Fig. S2*B*) sensitivity in CBS138 and Indian clinical strains, albeit to different extent. These results suggest that CgSet4-dependent control of antifungal resistance is common among clinical strains of *C. glabrata*.

**Figure S3:**
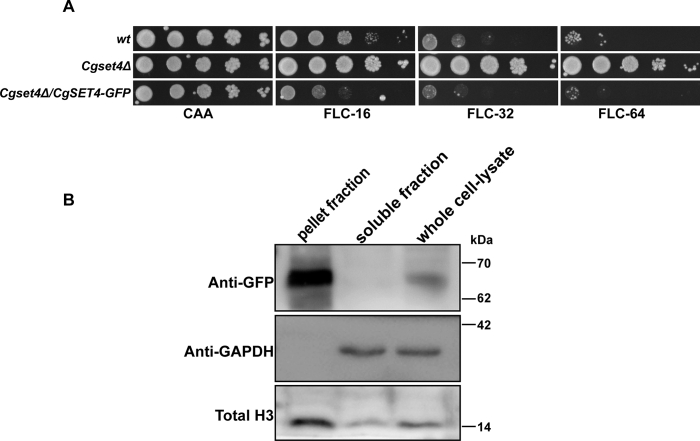
**CgSet4-GFP complementsdecreased fluconazole susceptibility of the *Cgset4Δ* mutant.**

Next, to delineate the role of CgSet4 in antifungal resistance, we decided to first check its cellular localization. For this, we generated CgSet4-GFP fusion protein and found it to be functional, as it complemented FLC resistance phenotype of the *Cgset4Δ* mutant (Fig. S3*A*). Next, the confocal imaging analysis revealed that CgSet4 is primarily localized to the nucleus under all (regular, FLC-treated, and CSP-treated) conditions (Fig. 2*C*), indicating a predominant nuclear role for CgSet4 in *C. glabrata*. Furthermore, CgSet4-GFP was found to be highly enriched in the insoluble chromatin fraction, whereas being totally absent in the soluble cytosolic fraction (Fig. S3*B*), indicating that CgSet4 is a chromatin-associated protein.

Since *CgSET4* gene loss was associated with drug resistance, we next checked the effect of antifungal treatment on *CgSET4* transcript and protein levels. For this, we compared *CgSET4* transcript and protein levels, *via* quantitative RT–PCR (qRT–PCR) and Western analysis, respectively, between untreated and FLC- or CSP-treated *wt* cells (Fig. 2*D*). We found that *CgSET4* transcript (Fig. 2*D*) and CgSet4-GFP (∼58 kDa band) protein (Fig. 2*E*) levels were about two- to five-fold lower in FLC- and CSP-treated *wt* cells, compared with untreated *wt* cells, indicating that *C. glabrata* responds to azole and echinocandin exposure by downregulating *CgSET4* expression. Of note, this antifungal-induced *CgSET4* repression is in accordance with the FLC and CSP resistance observed in the *Cgset4Δ* mutant (Fig. 1, *A* and *D*). Collectively, our data suggest that CgSet4 is a nuclear protein that may be a pivotal component of the azole and echinocandin antifungal resistance regulatory system in *C. glabrata*.

### CgSet4 negatively regulates the *CgPDR1* regulon

**Figure 3:**
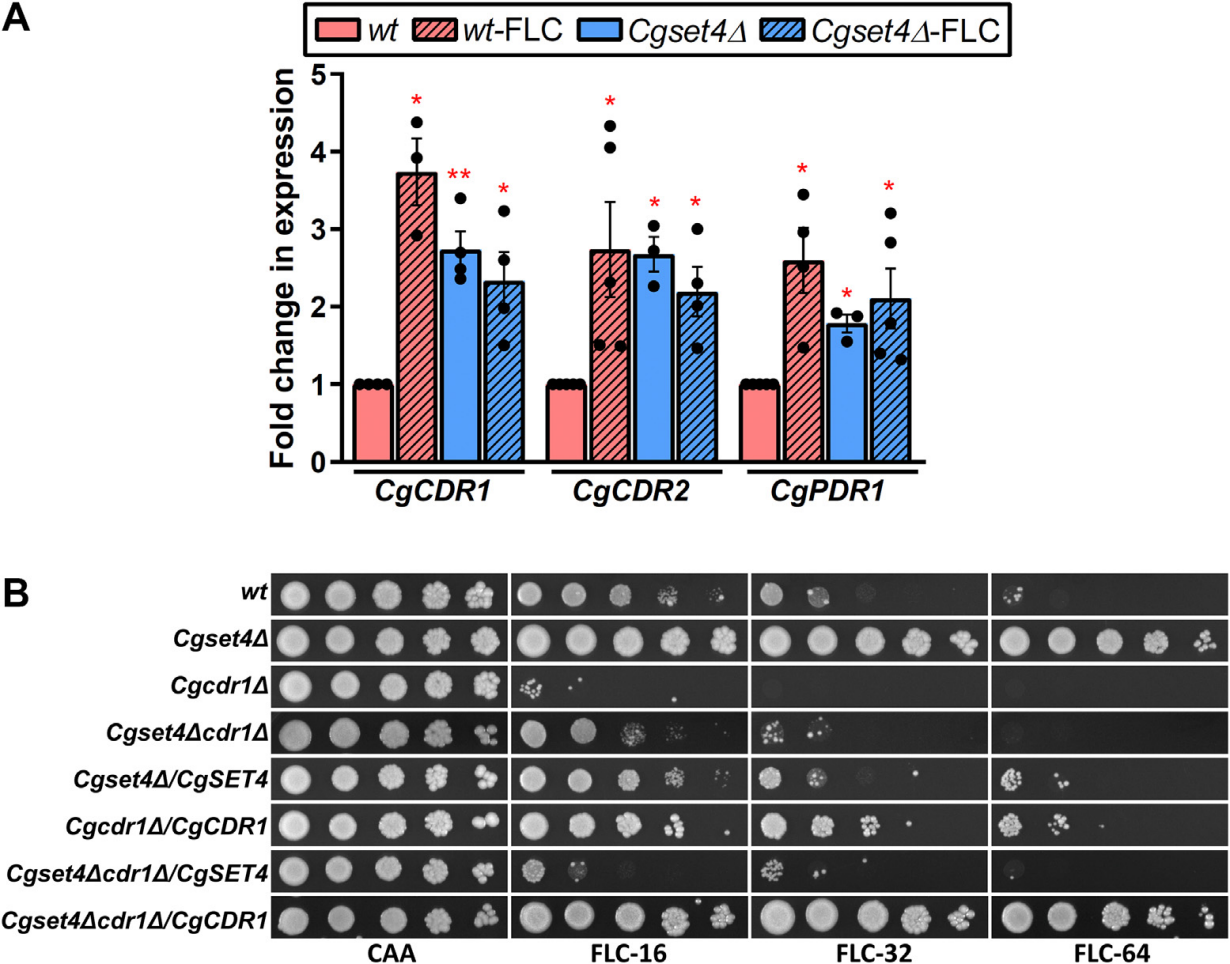
**CgSet4 is a negative regulator of the *CgPDR1* regulon**. *A*, qRT–PCR analysis showing elevated transcript levels of *CgCDR1*, *CgCDR2*, and *CgPDR1* genes in the *Cgset4Δ* mutant. Log-phase *wt* and *Cgset4Δ* cells were left untreated or treated with 64 μg/ml fluconazole (FLC) in CAA medium for 1 h, and transcript levels of indicated genes were determined by qRT–PCR. Data (mean ± SEM, n = 3–5) were normalized against the *CgACT1* mRNA control, and represent fold change in gene expression in FLC-treated *wt*, *Cgset4Δ*, and FLC-treated *Cgset4Δ* cells, as compared with CAA medium–grown *wt* cells (taken as 1.0). ∗*p* < 0.05; ∗∗*p* < 0.01, paired two-tailed Student's *t* test. *B*, serial dilution spotting analysis illustrating FLC susceptibility of indicated *Candida glabrata* strains. FLC was used at a concentration of 16 μg/ml (FLC-16), 32 μg/ml (FLC-32), and 64 μg/ml (FLC-64) in CAA medium. CAA, casamino acid; qRT–PCR, quantitative RT–PCR.

Since the azole resistance in *C. glabrata* is primarily associated with overexpression of the multidrug transporter–encoding genes *CgCDR1* and *CgCDR2*, owing to increased protein or activity levels of CgPdr1 (14), we next checked the transcript levels of *CgPDR1*, *CgCDR1*, and *CgCDR2* genes in *Cgset4Δ* mutant. We found that 1.8-, 2.7-, and 2.7-fold increased expression of *CgPDR1*, *CgCDR1*, and *CgCDR2* genes, respectively, in the *Cgset4Δ* mutant (Fig. 3*A*). Furthermore, while FLC exposure resulted in elevated *CgPDR1*, *CgCDR1*, and *CgCDR2* transcript levels in *wt* cells, no such increase was observed in FLC-treated *Cgset4Δ* cells, compared with untreated *Cgset4Δ* cells (Fig. 3*A*). These data suggest that FLC resistance in the *Cgset4Δ* mutant could be due to high basal level expression of *CgPDR1* regulon genes.

To verify this notion, we deleted *CgCDR1* gene from the genome of both *wt* and *Cgset4Δ* strains. We found that while *CgCDR1* gene loss rendered *wt* cells highly susceptible to FLC, *CgCDR1* gene loss in the *Cgset4Δ* mutant reversed the azole resistance phenotype of the mutant, as the double mutant *Cgset4Δcdr1Δ* exhibited *wt*-like sensitivity to FLC (Fig. 3*B*). Importantly, expression of *CgSET4* and *CgCDR1* genes in the *Cgset4Δcdr1Δ* mutant led to elevated and diminished FLC susceptibility, similar to *Cgcdr1Δ* and *Cgset4Δ* mutant, respectively (Fig. 3*B*). Of note, the *wt*-like and not the *Cgcdr1Δ-*like FLC susceptibility of the double mutant *Cgset4Δcdr1Δ* raises the possibility that genes other than *CgCDR1* may also contribute to FLC resistance in the *Cgset4Δ* mutant. Despite multiple attempts, we could not generate the double deletion strain *Cgset4Δpdr1Δ* lacking both *CgSET4* and *CgPDR1* genes, the reason underlying this is yet to be determined. Collectively, besides showing that FLC resistance in the *Cgset4Δ* mutant is predominantly dependent upon CgCdr1, these results implicate CgSet4 in the transcriptional repression of *CgPDR1* in *C. glabrata*.

### Cell wall composition is not altered in the *Cgset4Δ* mutant

**Figure S4:**
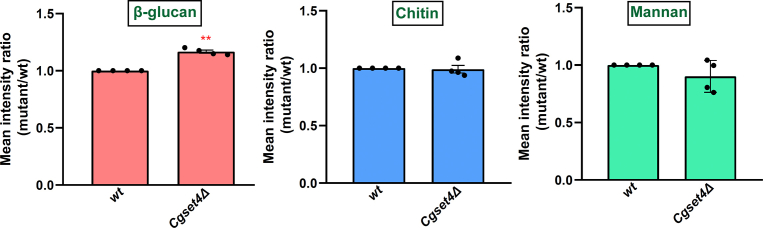
**Flow cytometry-based measurement of cell wall components.**

After elucidating the molecular basis of azole resistance, we next focused on the echinocandin resistance in the *Cgset4Δ* mutant. CSP inhibits synthesis of the cell wall component, β-1,3 d-glucan, and a compensatory increase in cell wall chitin levels has been reported upon CSP exposure (15, 26, 41). Therefore, we next checked if CSP resistance in *Cgset4Δ* mutant could be due to an altered cell wall composition in the mutant. For this, we determined levels of all three components of the cell wall, *viz*., β-glucan, mannan, and chitin, *via* aniline blue, concanavalin A, and calcofluor white–based cell staining assays, respectively. Fluorescence-activated cell sorting analysis showed similar mannan and chitin content in cell walls of the *wt* and *Cgset4Δ* strains, and a very mild increase in β-glucan levels in the mutant cell wall, as compared with the *wt* cell wall (Fig. S4), thereby ruling out any significant contribution of the cell wall components to CSP resistance in the *Cgset4Δ* mutant.

### Ergosterol biosynthesis gene expression is downregulated in response to CSP exposure

**Figure S5:**
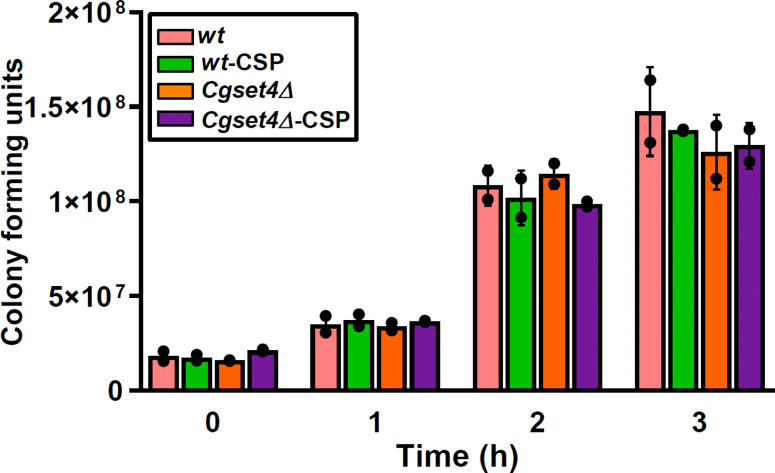
**Colony-forming unit assay-based viability analysis.**

CgSet4 was found to be associated with chromatin (Fig. S3*B*). Therefore, to uncover the molecular basis of CSP resistance in the *Cgset4Δ* mutant, we next profiled the global transcriptomes, using RNA-Seq approach, of logarithmic phase *wt* and *Cgset4Δ* cells in the presence and absence of CSP. For this, *C. glabrata* cells were treated with CSP at a sublethal concentration (0.25 μg/ml) for 1 h, and both *wt* and *Cgset4Δ* cells were found to retain viability during this treatment period (Fig. S5).

**Figure 4:**
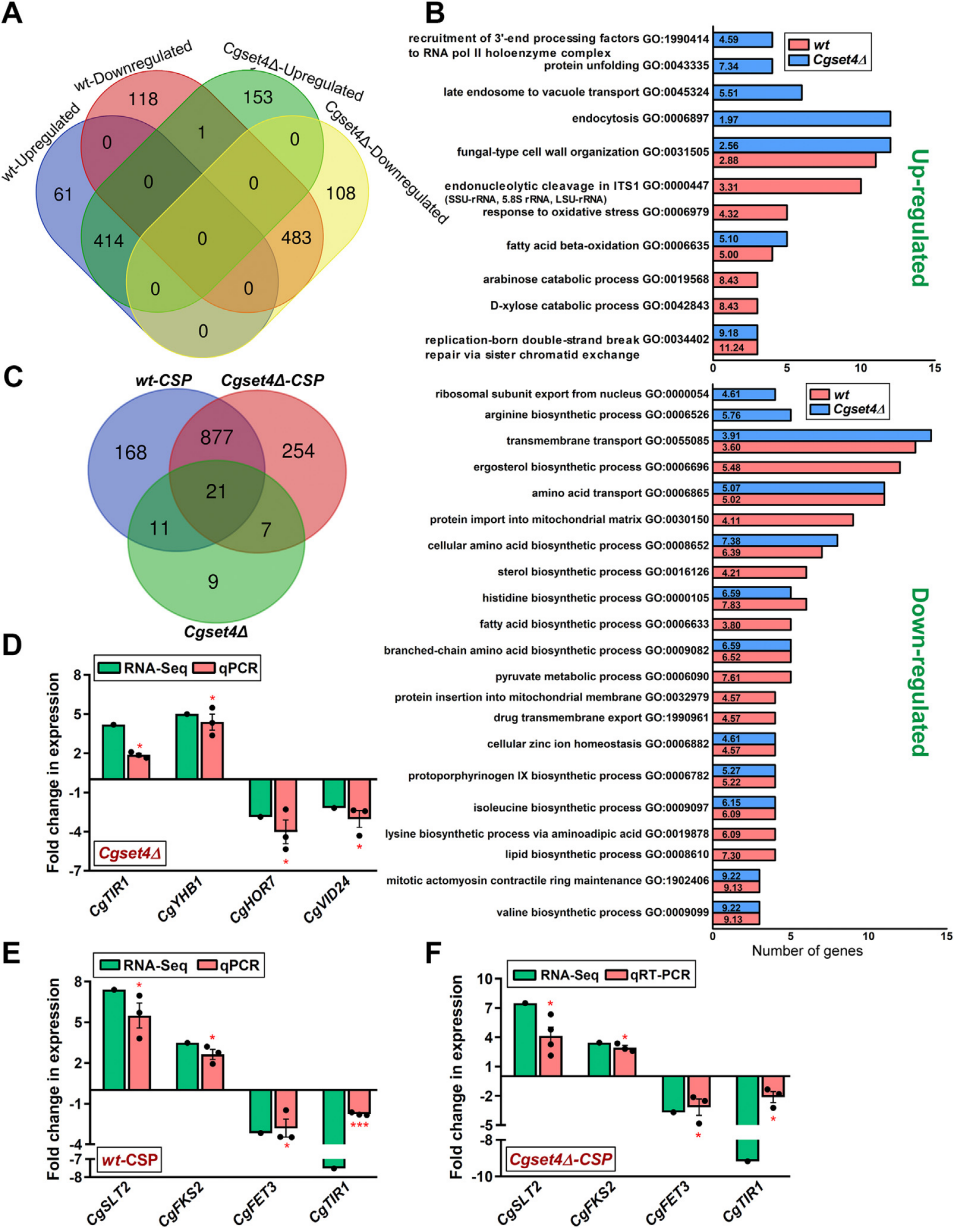
**Ergosterol biosynthesis genes are downregulated upon caspofungin (CSP) exposure.***A*, a Venn diagram illustrating overlap in upregulated and downregulated genes between CSP (250 ng/ml)-treated *wt* and *Cgset4Δ* strains. *B*, enriched Gene Ontology terms for Biological Process (*p* < 0.05) in differentially expressed genes in CSP-treated *wt* and *Cgset4Δ* strains, as determined using the DAVID tool. The fold-enrichment values are indicated in the individual annotation term bar. *C*, a Venn diagram illustrating overlap in differentially expressed genes among *Cgset4Δ* and CSP-treated *wt* and *Cgset4Δ* strains. *D*–*F*, qRT–PCR analysis validating the RNA-Seq data. Log-phase *wt* and *Cgset4Δ* cells were left untreated or treated with 250 ng/ml CSP in CAA medium for 1 h, and transcript levels of indicated genes were determined by qRT–PCR. Data (mean ± SEM, n = 3–4) were normalized against the *CgTDH3* mRNA control, and represent fold change in expression of indicated genes in the CAA medium–grown *Cgset4Δ* mutant as compared with the CAA medium–grown *wt* strain (taken as 1.0) (*D*), CSP-treated *wt* as compared with CAA medium–grown *wt* cells (taken as 1.0) (*E*), and CSP-treated *Cgset4Δ* as compared with CAA medium–grown *Cgset4Δ* cells (taken as 1.0) (*F*). ∗*p* < 0.05; ∗∗∗*p* < 0.001, paired two-tailed Student's *t* test. CAA, casamino acid; DAVID, Database for Annotation, Visualization, and Integrated Discovery; qRT–PCR, quantitative RT–PCR.

RNA-Seq analysis revealed 1077 genes to be differentially expressed (≥1.5-fold change in expression, and a *q* value of ≤0.05) in *wt* cells upon exposure to CSP (Fig. 4*A* and Table S2). Among these differentially expressed genes (DEGs), 475 and 602 genes were upregulated and downregulated, respectively (Fig. 4*A* and Table S2). Gene Ontology enrichment analysis for biological process by the DAVID (Database for Annotation, Visualization, and Integrated Discovery; https://david.ncifcrf.gov/) tool revealed upregulation of genes involved in fungal-type cell wall organization, response to oxidative stress, fatty acid (FA) beta-oxidation, and the catabolism of 5-carbon sugars, xylose and arabinose (Fig. 4*B* and Table S3*A*). Importantly, genes belonging to ergosterol biosynthesis, amino acid transport, zinc ion homeostasis, and amino acid biosynthetic process were found to be downregulated in CSP-treated *wt* cells, compared with untreated *wt* cells (Fig. 4*B* and Table S3*B*). Consistent with our qRT–PCR data (Fig. 2*D*), the *CgSET4* gene was downregulated in response to CSP exposure (Table S3*B*). Of note, CSP treatment also led to the repression of master regulators of the ergosterol biosynthesis genes, *CAGL0C01199g* (*CgUPC2A*) and *CAGL0F07865g* (*CgUPC2B*), and the plasma membrane sterol transporter gene (*CgAUS1*), and upregulation of the negative regulator of *ERG* gene biosynthesis *CAGL0D05434g* (*CgROX1*), and glycosylphosphatidylinositol-anchored aspartyl protease genes (*CgYPS1*, *CgYPS7*, *CgYPS6*, and *CgYPS10*) (Table S2). These proteases have previously been implicated in maintenance of the cell wall integrity, with CgYps1 also contributing to CSP tolerance (42, 43), whereas CgRox1, a heme-dependent repressor of the hypoxic genes, has recently been implicated in negative regulation of the *CgERG* genes (44).

Overall, our transcriptome data show an overlap with the previously reported transcriptomes of CSP-treated *C. albicans* and *S. cerevisiae* cells (41, 45, 46). For example, genes related to cell wall biogenesis and stress response, *viz*., *BAG7*, *CHS1*, *CWP1*, *FKS2*, *RLM1*, and *SLT2*, were upregulated, and genes involved in amino acid biosynthesis, *viz*., *ARG3*, *ILV6*, and *LEU1*, were downregulated in both *C. glabrata* and *S. cerevisiae* upon CSP exposure (Table S2 and (41, 45)). Similarly, genes involved in ergosterol biosynthesis, *ERG3*, *ERG4*, *ERG25*, and *ERG26*, and high-affinity iron transport, *FET3*, *FTR1*, and *FTH1*, were downregulated in both *C. glabrata* and *C. albicans* (Table S2 and (46).

Furthermore, despite CgSet4 being a chromatin-associated protein, its loss did not have a big impact on the transcriptome of *C. glabrata*, and only 48 genes were found to be differentially expressed in the *Cgset4Δ* mutant, compared with *wt* cells (Fig. 4*C* and Table S4). Of 48 genes, 24 were upregulated and 24 were downregulated in the *Cgset4Δ* mutant (Table S4). Of these gene sets, 7 and 10 genes were upregulated and downregulated, respectively, upon both deletion of *CgSET4* gene and CSP treatment of *wt* cells (Tables S2 and S4). Furthermore, three genes were upregulated upon CSP treatment of *wt* cells, whereas these were downregulated upon *CgSET4* deletion (Tables S2 and S4). These genes included *CgARO10*, *CgHBT1*, and *CgHOR7* (Tables S2 and S4). Similarly, 12 genes were downregulated in response to CSP treatment of *wt* cells, whereas these were upregulated upon *CgSET4* deletion, which also included four ergosterol biosynthesis genes, *CgERG2*, *CgERG3*, *CgERG5*, and *CgERG11* (Tables S2 and S4). DAVID analysis revealed genes involved in ergosterol biosynthesis, sterol import, and carbohydrate transport to be upregulated (Table S5*A*), whereas genes involved in the inositol biosynthetic process were found to be downregulated, upon *CgSET4* deletion in *C. glabrata* (Table S5*B*). Of note, *CgERG2*, *CgERG3*, *CgERG5*, *CgERG11*, *CgAUS1*, and *CgROX1* genes were upregulated in *Cgset4Δ* cells, compared with *wt* cells (Table S4).

**Figure S6:**
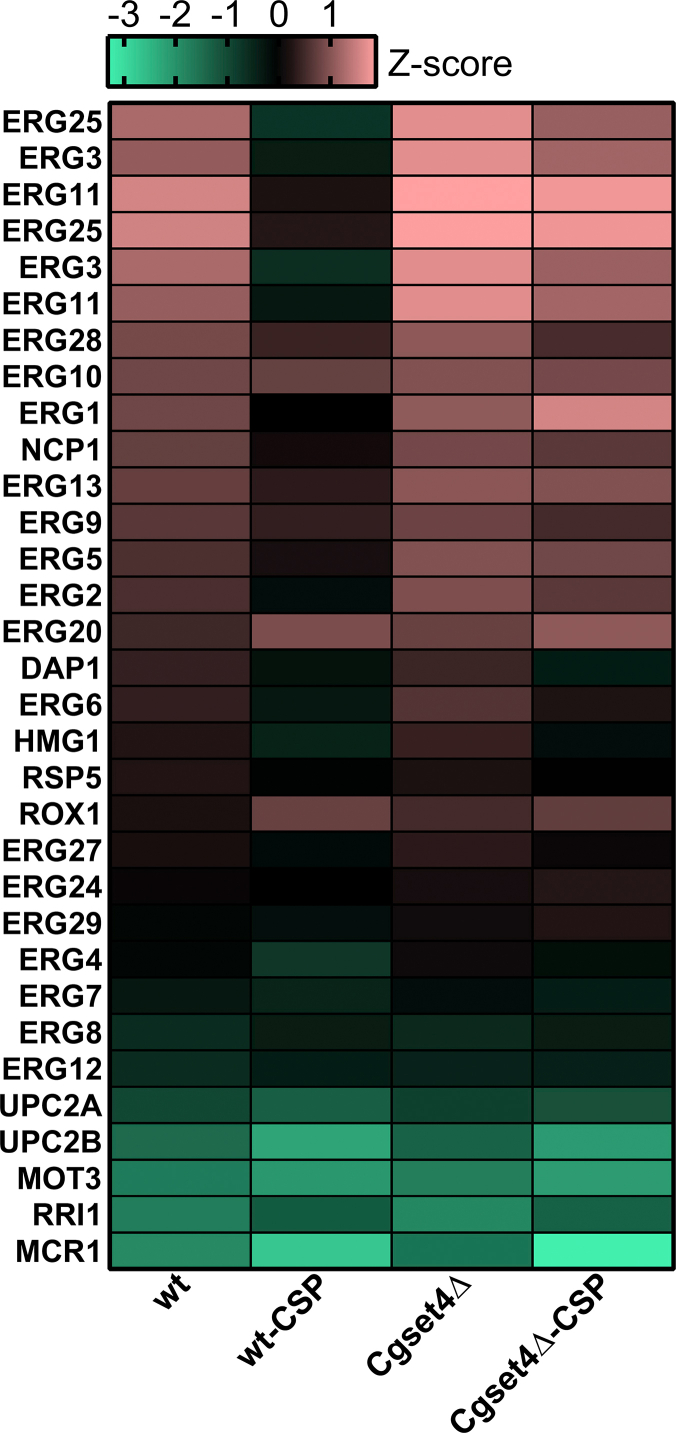
**Heat map illustrating expression of genes of the ergosterol biosynthesis pathway in indicated strains, as determined by RNA-Seq.**

Notably, CSP exposure led to differential regulation of 1159 genes in the *Cgset4Δ* mutant, compared with untreated *Cgset4Δ* cells, with 568 and 591 displaying upregulation and downregulation, respectively (Fig. 4*A* and Table S6). Of these, 414 upregulated genes and 483 downregulated genes were common between CSP-treated *wt* and *Cgset4Δ* cells (Fig. 4*A* and Table S6). Gene Ontology analysis revealed a substantial overlap (898 genes) between CSP-treated *wt* and *Cgset4Δ* cells (Fig. 4*C*, Tables S3 and S7), with fungal-type cell wall organization, FA beta-oxidation, endocytosis, and protein unfolding genes displaying upregulation in CSP-treated *Cgset4Δ* cells (Fig. 4*B* and Table S7*A*), and amino acid transport, zinc ion homeostasis, ribosomal subunit export from nucleus, and cellular amino acid biosynthetic process genes showing downregulation in CSP-treated *Cgset4Δ* cells (Fig. 4*B* and Table S7*B*). Of note, while CSP exposure led to wholesale downregulation of 14 ergosterol biosynthesis genes in *wt* cells (Table S2), its negative regulatory effect in *Cgset4Δ* cells was seen for five ergosterol biosynthesis genes, *CgERG3*, *CgERG11*, *CgERG26*, *CgERG28*, and *CgHMG1*, which were downregulated in the mutant (Table S4). Expression of the ergosterol biosynthesis pathway genes in untreated- and CSP-treated *wt* and *Cgset4Δ* strains is schematically presented in Fig. S6. Furthermore, similar to *wt* cells, CSP treatment led to upregulation of three glycosylphosphatidylinositol-anchored aspartyl protease genes, *CgYPS1*, *CgYPS7*, and *CgYPS10*, in *Cgset4Δ* cells (Table S6). Altogether, the transcriptional profiling data suggest that *C. glabrata* responds to echinocandin stress by transcriptional reprogramming of the cell wall organization, ergosterol biosynthesis, sugar metabolism, amino acid biosynthesis and transport, zinc ion homeostasis, and oxidative stress response genes.

Next, we verified the RNA-Seq data for many genes by qRT–PCR analysis and found good agreement between the RNA-Seq and qRT–PCR datasets. First, we showed that *CgSET4* disruption resulted in transcriptional activation of *CgYHB1* and *CgTIR1* genes and repression of *CgHOR7* and *CgVID24* genes (Fig. 4*D* and Table S4). Next, we showed that *CgSLT2* and *CgFKS2* genes, and *CgFET3* and *CgTIR1* genes, were upregulated and downregulated, respectively, in CSP-treated *wt* (Fig. 4*E*) and *Cgset4Δ* (Fig. 4*F*) cells. Of note, while CgSlt2 is the terminal mitogen-activated protein kinase of the cell wall integrity pathway, and has previously been implicated in CSP tolerance (47, 48), CgFks2 is the 1,3-β-d-glucan synthase enzyme, which is required for β-glucan synthesis, and mutations in the *CgFKS2* gene have been associated with CSP resistance in *C. glabrata* (15, 25).

Collectively, our transcriptome data suggest that expression of the ergosterol biosynthesis genes is altered in response to both *CgSET4* deletion and CSP exposure, underscoring a hitherto unknown effect of CSP treatment on ergosterol biosynthesis in *C. glabrata*. Of note, the CSP-induced transcriptional downregulation of the *CgSET4* gene in our RNA-Seq data is consistent with the CSP resistance phenotype of the *Cgset4Δ* mutant.

### CgSet4 negatively regulates basal *ERG* gene expression

Ergosterol is a major sterol in the plasma membrane and represents an integral cell membrane component, whose synthesis in the cell is regulated by oxygen and iron abundance (49, 50). Notably, ergosterol biosynthesis genes were upregulated in *Cgset4Δ* mutant (Table S4), with the mutant also exhibiting high level of resistance to both azole and echinocandin drugs (Fig. 1, *A* and *B*). In addition, CSP exposure resulted in downregulation of the *ERG* genes in *wt* cells (Table S2). Therefore, we hypothesized that besides impacting FLC resistance, ergosterol levels may also control resistance to the cell wall–targeting drug, CSP, in the *Cgset4Δ* mutant.

**Figure 5:**
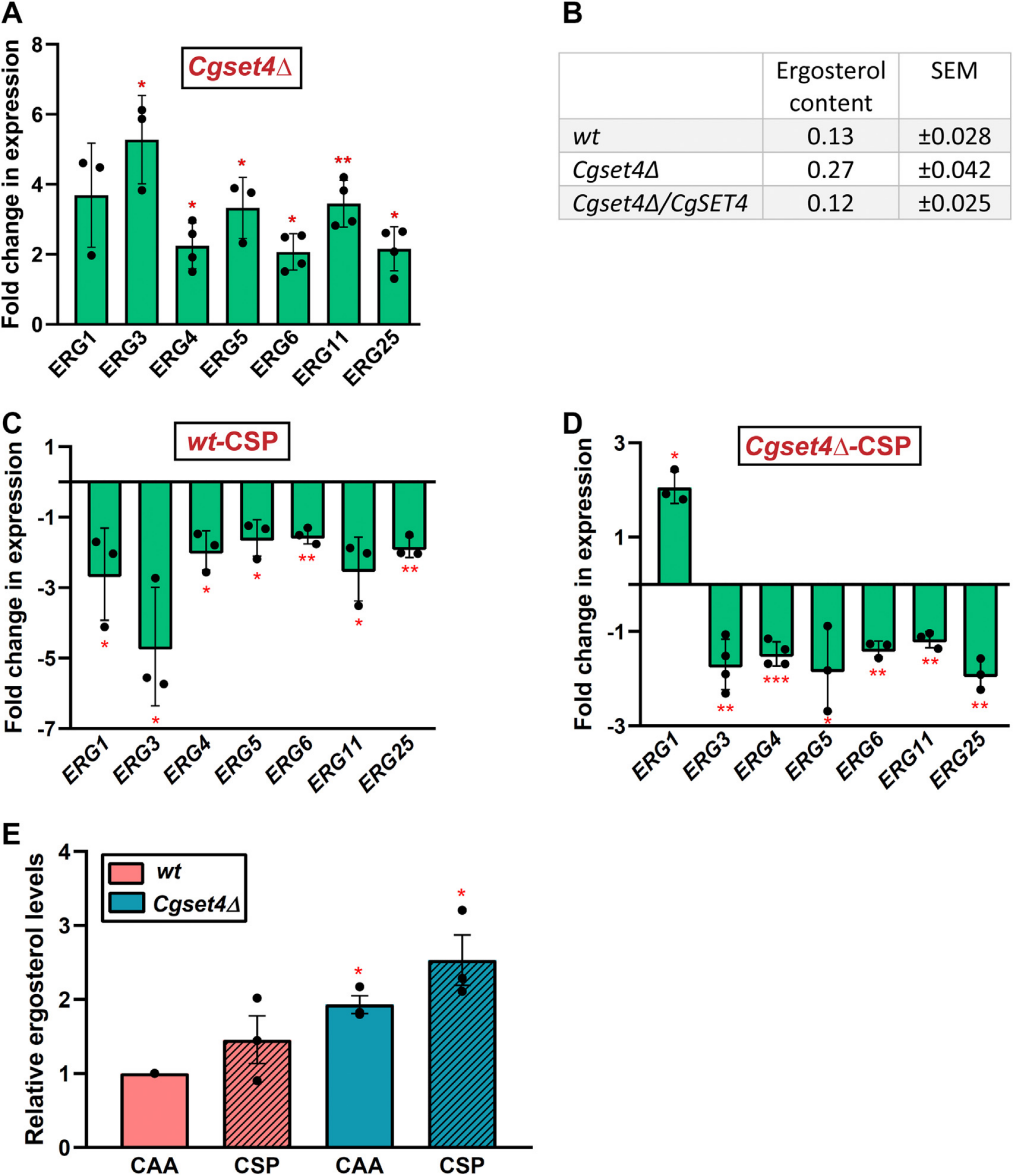
**CgSet4 is a negative regulator of the ergosterol biosynthesis pathway.***A*, qRT–PCR analysis showing expression of indicated *ERG* genes in log-phase *Cgset4Δ* cells. Data (mean ± SEM, n = 3–4) were normalized against the *CgACT1* mRNA control, and represent fold change in gene expression in the CAA medium–grown *Cgset4Δ* mutant as compared with the CAA medium–grown *wt* strain (taken as 1.0). ∗*p* < 0.05; ∗∗*p* < 0.01, paired two-tailed Student's *t* test. *B*, UV spectrophotometry–based measurement of ergosterol content of indicated log-phase *Candida glabrata* strains. Cultures were grown in CAA medium to log phase and collected. After PBS washes, sterols were extracted using *n*-heptane, and spectral profiles were recorded between 200 and 350 nm. The typical four-peak curve, indicating the presence of ergosterol and dehydroergosterol (DHE) in samples, was used to measure ergosterol content. Data (mean ± SEM, n = 3) indicate the percentage of ergosterol per gram of the wet weight of the cell pellet. *C* and *D*, qRT–PCR analysis showing expression of indicated *ERG* genes in caspofungin (CSP)-treated *wt* (*C*) and CSP-treated *Cgset4Δ* cells (*D*). Log-phase *wt* and *Cgset4Δ* cells were left untreated or treated with 250 ng/ml CSP in CAA medium for 1 h, and transcript levels of indicated genes were determined by qRT–PCR. Data (mean ± SEM, n = 3–4) were normalized against the *CgACT1* mRNA control, and represent fold change in gene expression in CSP-treated *wt* cells as compared with CAA medium–grown *wt* cells (taken as 1.0) (*C*), and CSP-treated *Cgset4Δ* cells as compared with CAA medium–grown *Cgset4Δ* cells (taken as 1.0) (*D*). ∗*p* < 0.05; ∗∗*p* < 0.01; ∗∗∗*p* < 0.001, paired two-tailed Student's *t* test. *E,* ergosterol content in log-phase cells of indicated *C. glabrata* strains, which were grown in the CAA medium lacking (CAA) or containing 250 ng/ml CSP for 1 h, was measured. Data (mean ± SEM, n = 3) represent fold change in ergosterol levels in indicated strains as compared with CAA medium–grown *wt* cells (considered as 1.0). ∗*p* < 0.05, paired two-tailed Student's *t* test. CAA, casamino acid; qRT–PCR, quantitative RT–PCR.

**Figure S7:**
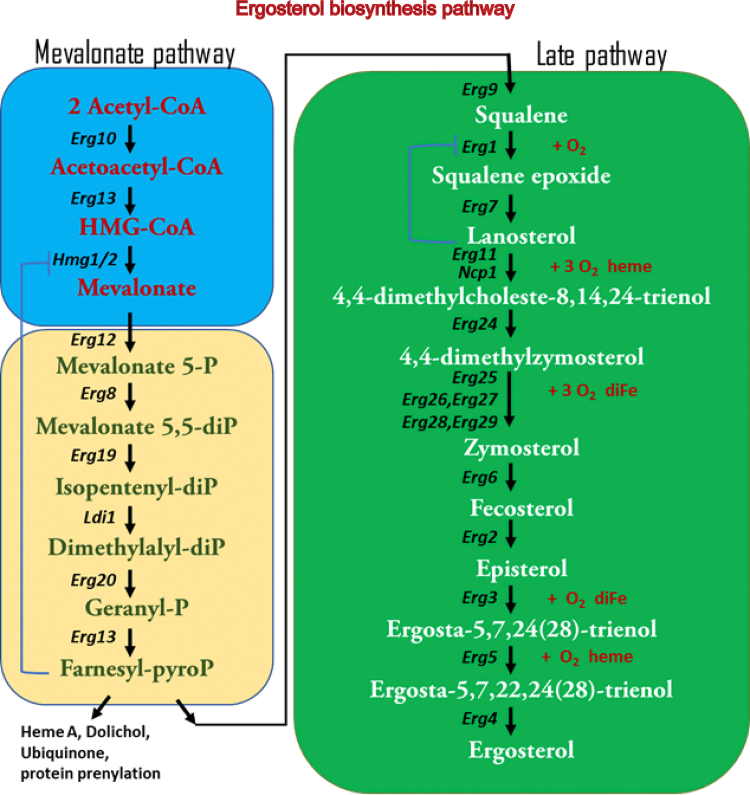
**A schematic representation of the ergosterol biosynthesis pathway in yeast.**

To test this hypothesis, we performed four experiments. First, we examined the expression of different *ERG* genes in the *Cgset4Δ* mutant. Ergosterol biosynthesis is a multistep process that consists of three modules (Fig. S7) (50). The mevalonate biosynthesis module takes place in the vacuole and the mitochondria, the farnesyl pyrophosphate biosynthesis pathway occurs in the vacuole, and the late stages of the pathway predominantly occur in the endoplasmic reticulum membrane (50, 51). The late pathway starts and ends with the formation of squalene and ergosterol, respectively (50, 51). Since late steps of the ergosterol biosynthesis pathway have been implicated in stress tolerance (50, 51, 52), we checked expression of the seven *ERG* genes, *CgERG1*, *CgERG3*, *CgERG4*, *CgERG5*, *CgERG6*, *CgERG11*, and *CgERG25*, which code for enzymes carrying out various reactions in the ergosterol biosynthesis pathway (Fig. S7). qRT–PCR analysis revealed about two- to five-fold higher expression of *CgERG1*, *CgERG3*, *CgERG4*, *CgERG5*, *CgERG6*, *CgERG11*, and *CgERG25* genes in the *Cgset4Δ* mutant, compared with *wt* cells (Fig. 5*A*), suggesting that *CgSET4* is required for repression of the late *ERG* genes under regular growth conditions. Of note, *CgERG3*, *CgERG5*, and *CgERG11* transcript levels were also higher in *Cgset4Δ* mutant, compared with *wt* cells, in our RNA-Seq analysis (Table S4 and Fig. S6).

Second, we measured total ergosterol levels in the *Cgset4Δ* mutant. Consistent with increased *ERG* gene expression in the *Cgset4Δ* mutant, we found two-fold higher ergosterol content in the mutant, compared with *wt* cells (Fig. 5*B*). Expression of the *CgSET4* gene led to *wt*-like ergosterol levels in the *Cgset4Δ* mutant (Fig. 5*B*), indicating that increased ergosterol content in the *Cgset4Δ* mutant was due to the lack of the *CgSET4* gene. Collectively, these results suggest that CgSet4 inhibits the ergosterol biosynthesis process *via* downregulation of the *CgERG* genes in *C. glabrata*.

Third, we verified the effect of *CgSET4* deletion on CSP-mediated downregulation of the ergosterol biosynthesis pathway, as RNA-Seq analysis had revealed *ERG* genes to be repressed in CSP-treated *wt* cells (Table S2 and Fig. S6). We observed that CSP treatment led to transcriptional repression of *CgERG3*, *CgERG4*, *CgERG5*, *CgERG6*, *CgERG11*, and *CgERG25* genes in both *wt* (Fig. 5*C*) and *Cgset4Δ* (Fig. 5*D*) strains. Importantly, however, *CgERG1* was found to be downregulated and upregulated in CSP-treated *wt* and *Cgset4Δ* cells, respectively (Fig. 5, *C* and *D*), indicating *CgSET4*-dependent transcriptional downregulation of the *CgERG1* gene in response to CSP exposure. Of note, *CgERG1* transcript levels were found to be increased upon *CgSET4* deletion (Fig. 5*A*), suggesting a negative regulatory role for CgSet4 in *CgERG1* expression. The molecular basis underlying CgSet4-dependent CSP-induced upregulation of *CgERG1*, which is the converse of that of the other *CgERG* genes, is yet to be determined. Altogether, our results suggest that CgSet4 is indispensable for the regulation of basal expression of *ERG* genes, but it has no prominent role in global downregulation of the ergosterol biosynthesis pathway under CSP-treated conditions.

Fourth, we measured ergosterol content in CSP-treated *wt* and *Cgset4Δ* cells. Contrary to expectations from the transcript profiling data, the CSP exposure did not result in a significant decrease in total ergosterol levels in *wt* cells (Fig. 5*E*). Instead, the ergosterol content was similar between untreated and CSP-treated *wt* cells (Fig. 5*E*). Similarly, ergosterol levels were similar between untreated and CSP-treated *Cgset4Δ* cells, with *CgSET4* loss resulting in 2.0-fold to 2.5-fold higher ergosterol in both the presence and the absence of CSP (Fig. 5*E*). These results suggest a complex multifaceted regulation of the ergosterol biosynthesis process in response to the antifungal CSP, wherein the decreased transcript levels of *ERG* genes do not translate into lower cellular ergosterol levels. Alternatively, it is possible that while transcriptional downregulation of the *ERG* genes is observed within 1 h CSP treatment, changes in the cellular ergosterol levels may become conspicuous after prolonged CSP treatment when cells had undergone a few divisions. This possibility warrants further investigations. In this context, it is noteworthy that deletion of the *CgUPC2A* gene, which codes for a major transcriptional activator of *ERG* genes, was found to have no effect on the ergosterol content (44).

### Deletion of *CgERG3* and *CgERG4* genes reverses FLC resistance in the *Cgset4Δ* mutant

**Figure 6:**
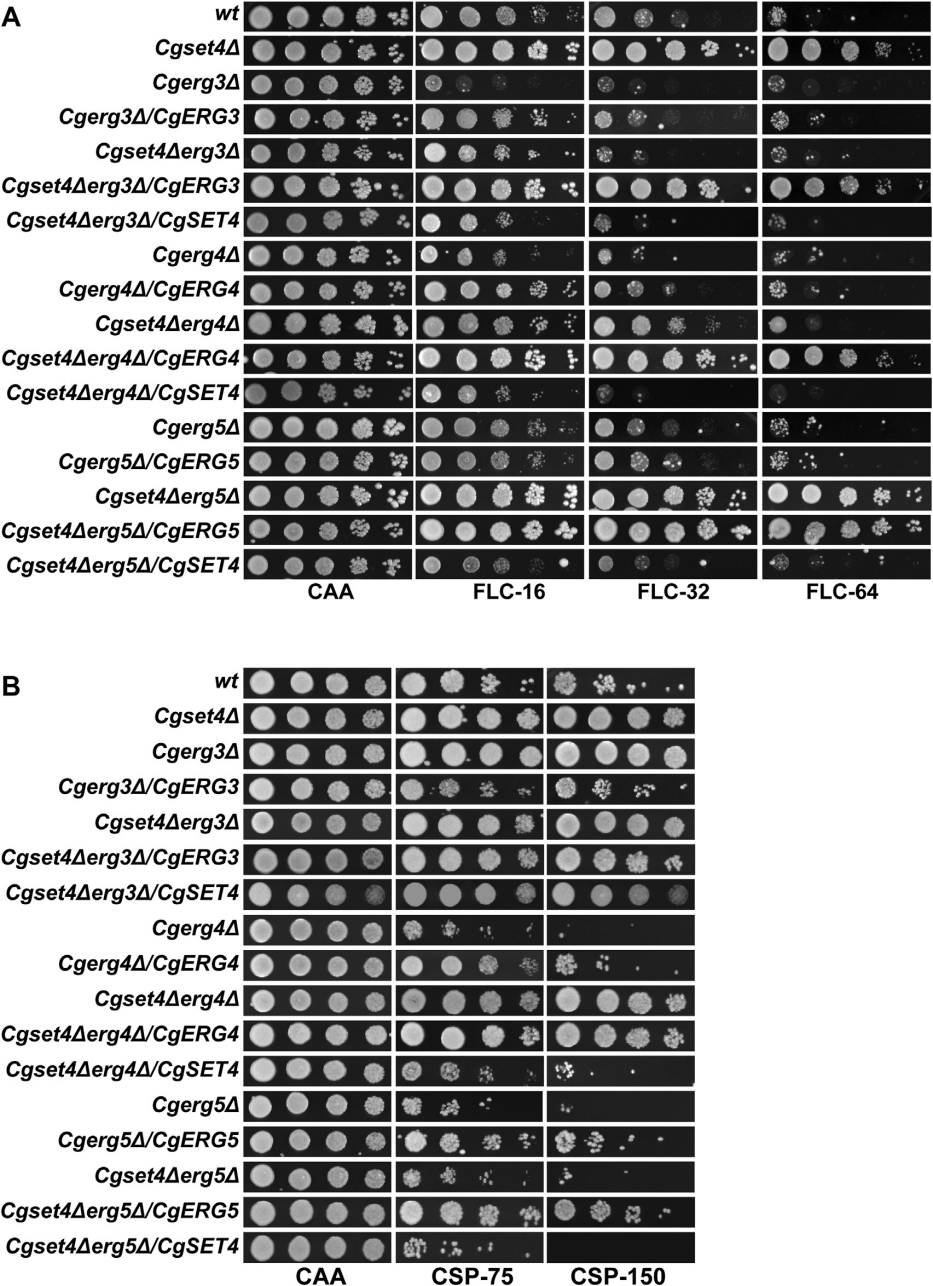
**Antifungal susceptibility of the *Cgset4Δ* mutant is modulated bythe*ERG* genes.***A,* serial dilution spotting analysis illustrating fluconazole (FLC) susceptibility of indicated *Candida glabrata* strains. FLC was used at a concentration of 16 μg/ml (FLC-16), 32 μg/ml (FLC-32), and 64 μg/ml (FLC-64) in CAA medium. B. Liquid medium–based growth analysis illustrating caspofungin (CSP) susceptibility of indicated *C. glabrata* strains. CSP was used at a concentration of 75 ng/ml (CSP-75) and 150 ng/ml (CSP-150) in CAA medium. CAA, casamino acid.

The late ergosterol biosynthesis pathway enzymes have previously been implicated in stress tolerance in *S. cerevisiae* (50, 51, 52). *CgERG3*, *CgERG5*, and *CgERG4* genes code for C-5 sterol desaturase, C-22 sterol desaturase, and C-24(28) sterol reductase enzymes, respectively, which catalyze last three steps of the ergosterol biosynthesis pathway (Fig. S7) (50). CgErg3, CgErg5, and CgErg4 produce ergosta-5,7,24(28)-trienol, ergosta-5,7,22,24(28)-trienol, and ergosterol, respectively (Fig. S7) (50, 51). Of note, the fungal Erg4 and Erg5 enzymes are not conserved in mammals (50), making them good antifungal targets. Importantly, mutations in the *CgERG3* gene have recently been found in both FLC and anidulafungin (an echinocandin drug)-resistant isolates of *C. glabrata* (53). Since the *Cgset4Δ* mutant displayed resistance to FLC as well as to CSP (Fig. 1, *A* and *D*), we next undertook a genetic approach to decipher CSP-dependent modulation of the ergosterol biosynthesis pathway, which may have an impact on azole response. For this, we generated double deletion strains, which lacked both *CgSET4*, and *CgERG3*, *CgERG5*, or *CgERG4* gene. As a control, the single deletion strains for *CgERG3*, *CgERG4*, and *CgERG5* genes were also created. Growth analysis in the presence of FLC revealed that CgErg3 and CgErg4 are required for FLC tolerance, as *Cgerg3Δ* and *Cgerg4Δ* mutants displayed attenuated growth on FLC-supplemented medium (Fig. 6*A*). Of note, *CgERG3* deletion has previously been associated with increased azole susceptibility (54). In contrast, *CgERG5* gene loss had no effect on FLC susceptibility of *C. glabrata* (Fig. 6*A*). Ectopic expression of *CgERG3* and *CgERG4* genes complemented the elevated FLC susceptibility of *Cgerg3Δ* and *Cgerg4Δ* mutants, respectively (Fig. 6*A*), suggesting that the azole sensitivity is due to the lack of the corresponding *CgERG* gene. Furthermore, the deletion of *CgERG3* and *CgERG4* genes in the *Cgset4Δ* mutant background reversed the FLC resistance phenotype of the *Cgset4Δ* mutant (Fig. 6*A*), raising the possibility of an essential requirement for these genes as the downstream target/effector of CgSet4-dependent FLC response. Notably, similar to the *Cgset4Δ* mutant, the *Cgset4Δerg5Δ* double mutant was found to be FLC resistant (Fig. 6*A*), indicating a dispensable role for CgErg5 in CgSet4-dependent modulation of the azole response. Moreover, since the *Cgerg5Δ* mutant did not exhibit elevated FLC susceptibility, it is possible that CgErg5 functions in ergosterol biosynthesis can either be bypassed and/or performed by another enzyme of the pathway. Alternatively, the differential FLC susceptibility of *Cgerg3Δ*, *Cgerg4Δ*, and *Cgerg5Δ* mutants could be due to impairment in both ergosterol biosynthesis and functioning of multidrug efflux pumps. In this context, it is noteworthy that the activity of multidrug transporters is known to be affected by the membrane lipid environment, and the plasma membranes of *S. cerevisiae erg3Δ*, *erg4Δ*, and *erg5Δ* mutants have been reported to be hyperpolarized, hyperpolarized, and nonhyperpolarized, respectively (55).

### Deletion of *CgERG5* reverses CSP resistance in the *Cgset4Δ* mutant

We next examined mutants’ growth in the presence of CSP. We found that while deletion of *CgERG4* and *CgERG5* genes rendered *C. glabrata* cells susceptible to CSP, *CgERG3* gene loss led to CSP resistance (Fig. 6*B*). These results indicate opposite roles for *CgERG3* and *CgERG4* and *CgERG5* genes in CSP susceptibility in *C. glabrata*. Of note, *CgERG5* deletion has earlier been reported to result in CSP sensitivity (56). Interestingly, CSP resistance in the *Cgset4Δ* mutant was not observed upon deletion of the *CgERG5* gene, with the *Cgset4Δerg5Δ* mutant in fact displaying increased CSP susceptibility, compared with *wt* cells (Fig. 6*B*). Contrarily, the *Cgset4Δerg3Δ* and *Cgset4Δerg4Δ* mutants exhibited increased growth in the presence of CSP, similar to that of the *Cgset4Δ* mutant (Fig. 6*B*). These data point toward CgSet4-dependent and CgSet4-independent requirement of *CgERG* genes in regulating antifungal resistance, with *CgERG4* loss resulting in increased susceptibility to both azole and echinocandin tolerance, and *CgERG3* and *CgERG5* loss leading to increased FLC and decreased CSP, and increased CSP sensitivity, respectively. These data suggest that the ergosterol biosynthesis pathway may be more closely linked with the action of cell wall–targeting echinocandin drugs, than it is currently being considered. In this context, it is worth noting that *Cgerg1Δ*, *Cgerg3Δ*, and *Cgerg11Δ* mutants have been shown to exhibit altered expression of *ERG* genes (23, 54). Therefore, it is possible that the differential expression of other *ERG* genes may contribute to distinct antifungal susceptibilities of double deletion mutants. However, the precise basis underlying the differential CSP susceptibilities of *CgergΔ* and *Cgset4ΔergΔ* mutants is yet to be determined.

Altogether, we infer from these data that the upregulated ergosterol biosynthesis process in the *Cgset4Δ* mutant contributes to elevated ergosterol content and may also modulate the response to CSP stress. Our data also raise a possibility of a possible nexus between CgSet4-dependent azole and echinocandin resistance and CgSet4-dependent negative regulation of *CgERG* gene expression.

### CgSet4 regulates ergosterol biosynthesis through CgUpc2a

**Figure 7:**
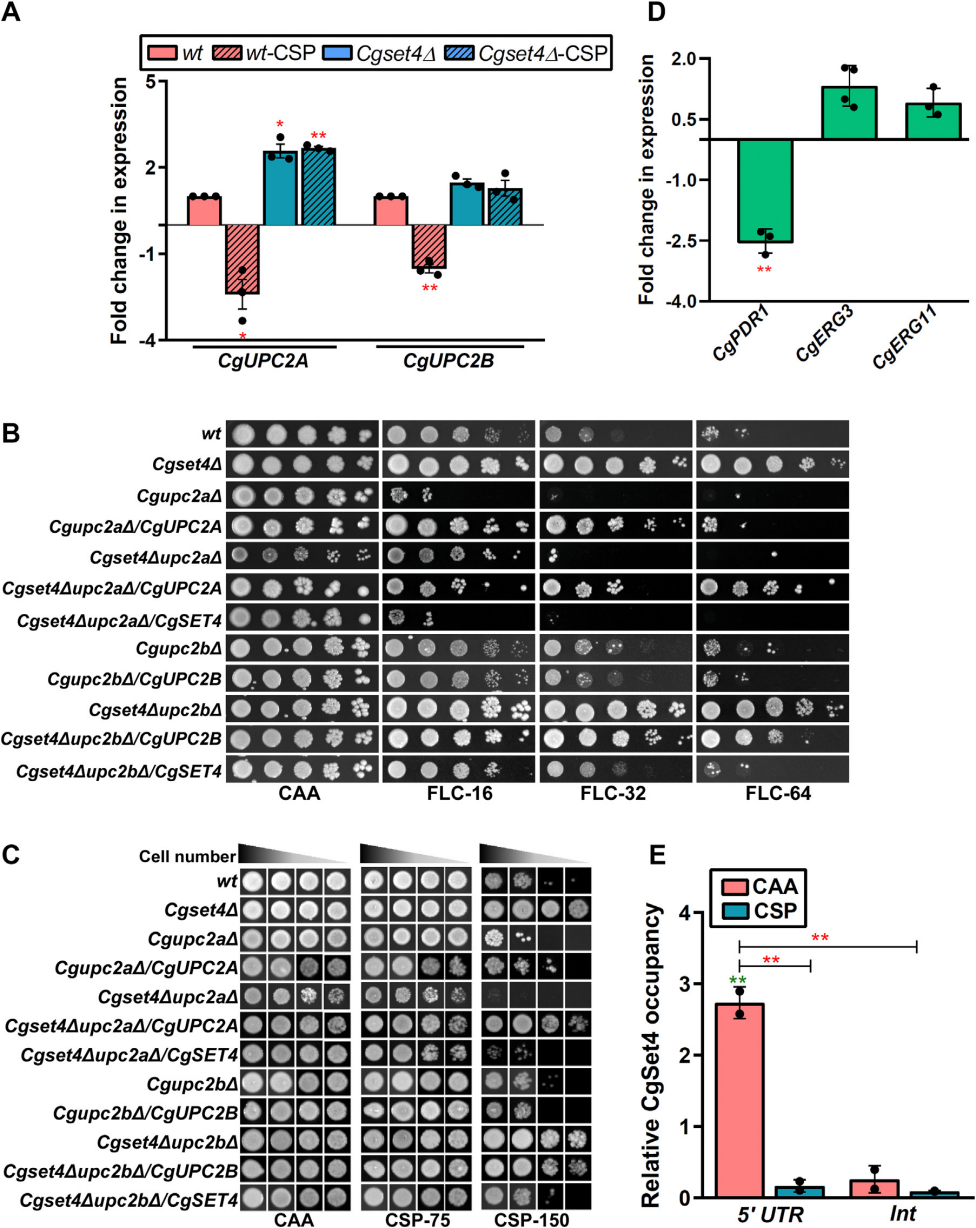
**CgUpc2A regulates caspofungin (CSP) susceptibility of the *Cgset4Δ* mutant.***A,* qRT–PCR analysis showing transcriptional downregulation of *CgUPC2A* and *CgUPC2B* genes in response to CSP exposure. Log-phase *wt* and *Cgset4Δ* cells were left untreated or treated with 250 ng/ml CSP in CAA medium for 1 h, and *CgUPC2A* and *CgUPC2B* transcript levels were determined by qRT–PCR. Data (mean ± SEM, n = 3) were normalized against the *CgTDH3* mRNA control and represent fold change in gene expression in indicated strains as compared with CAA medium–grown *wt* cells (taken as 1.0). ∗*p* < 0.05; ∗∗*p* < 0.01, paired two-tailed Student's *t* test. *B,* serial dilution spotting analysis illustrating fluconazole susceptibility of indicated *C. glabrata* strains. Fluconazole was used at a concentration of 16 μg/ml (FLC-16), 32 μg/ml (FLC-32), and 64 μg/ml (FLC-64) in CAA medium. *C,* liquid medium–based growth analysis illustrating CSP susceptibility of indicated *C. glabrata* strains. *C. glabrata* strains were cultured at 30 ^ο^C in CAA medium lacking (CAA) or containing 75 ng/ml (CSP-75) or 150 ng/ml (CSP-150) CSP for 16 h. After incubation, cultures were diluted in PBS, and 3 μl of undiluted, and 10-, 100-, and 300-fold diluted cultures were spotted on CAA medium, and growth was recorded after 1 day of growth at 30 °C. *D,* qRT–PCR analysis showing *CgPDR1*, *CgERG3*, and *CgERG11* transcript levels in *Cgset4Δupc2aΔ* mutant. Transcript levels were measured in YPD medium–grown, log-phase *wt*, and *Cgset4Δupc2aΔ* cells by qRT–PCR. Data (mean ± SEM, n = 3–4) were normalized against the *CgTDH3* mRNA control, and represent fold change in gene expression in the double mutant *Cgset4Δupc2aΔ* as compared with *wt* cells (taken as 1.0). ∗∗*p* < 0.01, paired two-tailed Student's *t* test. *E,* ChIP analysis, showing CgSet4-GFP enrichment on *CgUPC2A* promoter (5′UTR) under normal growth conditions and decreased enrichment upon CSP exposure, was performed with anti-GFP antibody to detect CgSet4-GFP. Log phase–grown *Cgset4Δ*/Vector and *Cgset4Δ*/*CgSET4-GFP* strains were either left untreated or treated with 250 ng/ml CSP in CAA medium for 1 h. The percentage of input was calculated for each IP and the ChIP amplification was normalized to the DNA input samples. Data (n = 2) represent CgSet4 occupancy in both untreated and drug-treated conditions, compared with *Cgset4Δ*/Vector samples. The primers used detected the promoter and the internal region of the *CgUPC2A* gene. ∗∗*p* < 0.01, paired two-tailed Student's *t* test. CAA, casamino acid; ChIP, chromatin immunoprecipitation; IP, immunoprecipitation; qRT–PCR, quantitative RT–PCR.

To investigate the molecular link between CgSet4-mediated repression of *CgERG* genes and CSP resistance, we focused on two genes, *CgUPC2A* and *CgUPC2B*, that code for two Zn_2_–Cys_6_ transcriptional activators of the ergosterol biosynthesis pathway in *C. glabrata* (21), and performed four experiments. First, we checked transcript levels of *CgUPC2A* and *CgUPC2B* genes in the *Cgset4Δ* mutant and found 2.3-fold higher and similar *CgUPC2A* and *CgUPC2B* gene expression, respectively, in the *Cgset4Δ* mutant, compared with *wt* cells (Fig. 7*A*). Furthermore, CSP exposure led to 2.4-fold and 1.5-fold downregulation of *CgUPC2A* and *CgUPC2B* genes, respectively, in *wt* cells (Fig. 7*A*). Contrarily, *CgUPC2A* expression remained the same between CSP-treated and untreated *Cgset4Δ* cells (Fig. 7*A*), suggesting that CgSet4 may be required to control *CgUPC2A* gene expression in response to CSP exposure. Of note, a small 1.4-fold decrease in *CgUPC2B* transcript levels was observed in CSP-treated *Cgset4Δ* cells, compared with untreated *Cgset4Δ* cells (difference not conspicuous in Figure 7*A*, as it depicts comparison with the untreated *wt* cells), thereby ruling out a major role of CgSet4 in CSP-induced *CgUPC2B* downregulation.

**Figure S8:**
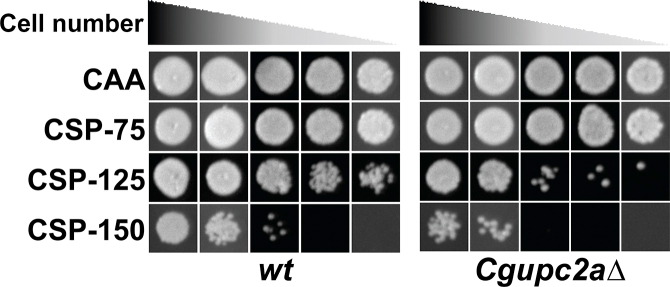
**Liquid medium-based growth analysis illustrating caspofungin susceptibility of the *Cgupc2aΔ* mutant.**

Second, we created deletion strains for *CgUPC2A* and *CgUPC2B* genes in both *wt* and *Cgset4Δ* strain backgrounds. Deletion of the *CgUPC2B* gene had no impact on the susceptibility toward FLC (Fig. 7*B*) or CSP (Fig. 7*C*). Contrarily, *CgUPC2A* gene loss rendered cells highly and moderately sensitive to FLC (Fig. 7*B*) and CSP (Figs. 7*C* and S8), respectively). These FLC susceptibility data are consistent with the published reports of CgUpc2a being a major regulator of ergosterol biosynthesis genes in *C. glabrata* under normal laboratory-growth conditions (21, 22). CgUpc2a has also been shown to be required for basal and FLC-induced transcription of *ERG* genes, with *CgUPC2A* deletion in an azole-susceptible dose-dependent and an azole-resistant clinical isolate leading to decreased ergosterol content (22). Of note, *CgUPC2A* gene loss has recently also been associated with CSP sensitivity (57).

**Figure S9:**
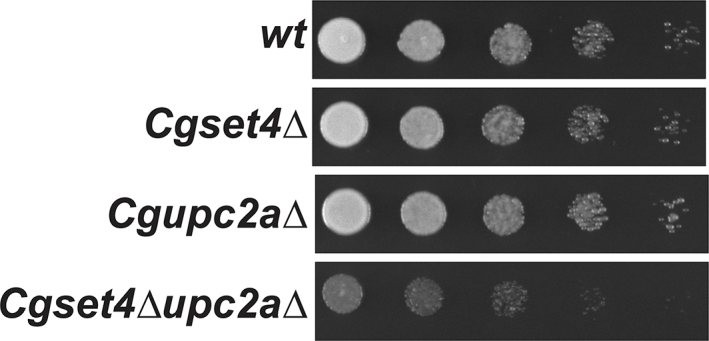
**Serial dilution spotting analysis illustrating attenuated growth of the *Cgset4Δupc2aΔ* mutant in YPD medium.**

Notably, the double mutant *Cgset4Δupc2bΔ* exhibited resistance to both FLC and CSP drugs, similar to the *Cgset4Δ* mutant (Fig. 7, *B* and *C*). Contrary to this, the *Cgset4Δupc2aΔ* double mutant was found to be sensitive to both antifungals, compared with the *Cgset4Δ* mutant (Fig. 7, *B* and *C*). Importantly, the *Cgset4Δupc2aΔ* double mutant displayed highly attenuated growth in rich yeast extract–peptone–dextrose (YPD) medium (Fig. S9), underscoring the stress that the simultaneous loss of both genes poses to the cellular machinery. Importantly, ectopic expression of *CgSET4*, *CgUPC2A*, and *CgUPC2B* genes in single and double mutants complemented the mutants’ altered drug susceptibility phenotypes (Fig. 7, *B* and *C*); however, the phenotypes were not rescued fully in *Cgset4Δupc2aΔ* and *Cgset4Δupc2bΔ* mutants expressing *CgUPC2A* and *CgUPC2B*, respectively (Fig. 7, *B* and *C*). The basis underlying this observation is yet to be determined.

Based on these data, which suggest that CgSet4 is likely to control ergosterol biosynthesis process by modulating the CgUpc2A-dependent *ERG* gene regulation, we hypothesized that the elevated expression of *CgUPC2A* and its target genes contribute to antifungal resistance in the *Cgset4Δ* mutant. If this is true, the *CgUPC2A* gene deletion is likely to impact the antifungal resistance gene expression negatively in the *Cgset4Δ* mutant. Therefore, to test this hypothesis, we checked the expression of *CgPDR1*, *CgERG3*, and *CgERG11* genes in the *Cgset4Δupc2aΔ* double mutant. We found two-fold lower, similar, and similar transcript levels of *CgPDR1*, *CgERG3*, and *CgERG11* genes, respectively, in the double mutant, compared with *wt* cells (Fig. 7*D*), indicating that CgUpc2A is a major activator of the *CgPDR1* gene in the *Cgset4Δ* mutant. Furthermore, the *wt*-like expression of *CgERG3* and *CgERG11* genes in the *Cgset4Δupc2aΔ* double mutant, in comparison to elevated *CgERG3* and *CgERG11* transcript levels in the *Cgset4Δ* mutant (Fig. 5*A*), implicates CgUpc2A in the activation of *CgERG* genes in the *Cgset4Δ* mutant.

Finally, to examine if CgSet4 directly regulates *CgUPC2A* expression, we performed the chromatin immunoprecipitation (ChIP) analysis using CgSet4-GFP, and found CgSet4 bound to the *CgUPC2A* promoter. Furthermore, CSP exposure led to a decrease in the occupancy of CgSet4 on the *CgUPC2A* promoter (Fig. 7*E*), which is consistent with a reduction in *CgSET4* transcript and protein levels in response to CSP exposure (Fig. 2, *C* and *D*). These data indicate multifactorial regulation of *CgUPC2A* gene expression, with probably another repressor accounting for the CSP-induced *CgUPC2A* downregulation. Notably, CgRox1 has recently been reported to be a negative regulator of *ERG* genes, with *Cgrox1Δupc2aΔ* mutant containing 1.5-fold higher ergosterol levels, indicating perturbed sterol homeostasis (44). Based on this report and the following three findings of our RNA-Seq analysis, we speculate that CgRox1 could play a role in *CgUPC2A* downregulation upon CSP exposure. First, the *Cgset4Δ* mutant was found to be quite proficient in repressing *CgERG* gene expression in response to CSP (Table S6 and Fig. S6); Second, *CgROX1* was upregulated in the *Cgset4Δ* mutant (Table S4 and Fig. S6). Third, *CgROX1* transcription is activated in CSP-treated *wt* cells (Table S2 and Fig. S6). However, the role of CgRox1 in cellular response to CSP is yet to be illustrated.

Collectively, we draw five major conclusions from our data. First, CgErg3, CgErg5, and CgErg4 enzymes modulate antifungal tolerance in *C. glabrata*. Second, CgSet4 is a negative regulator of *CgUPC2A* and *CgERG* gene expression under normal growth conditions. Third, *CgUPC2A* largely accounts for elevated levels of *CgPDR1* and *CgERG* transcripts in the *Cgset4Δ* mutant. Fourth, CSP exposure leads to transcriptional repression of *CgSET4*, *CgUPC2A*, and *CgUPC2B* genes, which may in turn probably contribute to the downregulated ergosterol biosynthesis pathway in CSP-treated *C. glabrata* cells. Finally, CgSet4 may not be a key regulator of the CgUpc2a gene regulatory circuit in the presence of CSP. Based on these findings, we propose that the complex transcriptional loops probably drive *CgERG* gene expression in *C. glabrata*.

Altogether, we show for the first time that the SET domain–containing protein CgSet4 is pivotal to antifungal resistance, as it acts as an upstream regulator of the CgUpc2a-dependent ergosterol biosynthesis gene expression system. In addition, our data suggest that ergosterol content in the fungal cell membrane may play a role in determining the efficacy of the cell wall–targeting antifungal drugs.

## Discussion

*C. glabrata* is a common cause of *Candida* bloodstream infections in patients with a weakened immune system (1). The successful treatment of *C. glabrata* infections is becoming increasingly difficult as two mainstream antifungal drugs, azoles and echinocandins, are proving to be less effective because of emerging resistance in *C. glabrata* isolates in hospitals (4, 5, 11, 12). Not only *C. glabrata* is intrinsically less susceptible to frequently used, cost-effective, and relatively safe azole antifungals, but it also acquires high levels of resistance to azole and echinocandin drugs, with the echinocandin class representing the last line of antifungal therapy in many hospitalized severely ill patients (4, 5, 9, 11, 12, 14, 15). The azole resistance in clinical settings has predominantly been attributed to gain-of-function mutations in the master transcriptional regulator-encoding gene, *CgPDR1*, whereas mutations in the hot-spot regions of β-1,3 glucan synthase enzyme–encoding genes *CgFKS1* and *CgFKS2* primarily account for echinocandin resistance in hospitals worldwide (14, 15). Chromatin architecture is pivotal to gene expression regulation, with histone post-translational modifications playing a key role in maintenance of chromatin homeostasis (58). The epigenetic control of antifungal resistance mechanisms in *C. glabrata* is beginning to be elucidated (28, 38, 56). Toward this end, we report an essential role for the SET domain–containing protein CgSet4 in the transcriptional downregulation of *CgPDR1* and *CgUPC2A* genes, which code for the master regulator of MDR and ergosterol biosynthesis genes, respectively, in *C. glabrata*. In addition, we present the first systematic analysis unveiling functions of CgSet1–CgSet6 and CgErg3–CgErg5 proteins in *C. glabrata*. We show that the last enzyme of the ergosterol synthesis pathway CgErg4 is required for both azole and echinocandin tolerance, and, of six SET domain–containing proteins, only CgSet4 acts as a negative regulator of both azole and echinocandin antifungal resistance.

The SET domain, that consists of strongly conserved sequence motif of about 130 amino acids, was initially identified in the *Drosophila* proteins, Su(var)3 to 9, Enhancer-of-zeste and Trithorax, which regulate gene expression during development (35, 59). The SET domain now has been reported in several proteins that perform diverse functions (30, 36, 37). The lysine methyltransferase activity has largely been associated with the SET domain–containing proteins, which modulate chromatin structure, function, and the consequent gene expression regulation (30, 36, 37). In addition to the SET domain, these proteins also possess cysteine-rich regions flanking N-terminal (pre-SET) and posterior to C-terminal (post-SET) region of the SET domain, which are pivotal to target protein recognition and lysine methyltransferase activity (36, 37).

The National Center for Biotechnology Information conserved domain search analysis (https://www.ncbi.nlm.nih.gov/Structure/cdd/wrpsb.cgi) revealed that the SET domain in CgSet4 belongs to the SET Superfamily cl40432. The *C. glabrata* Set4 protein is 350 amino acid-long, whereas Set4 in *S. cerevisiae* consists of 560 amino acids. Despite sharing 40% identity with CgSet4 protein, the *S. cerevisiae* Set4 could not complement the decreased FLC and CSP susceptibility of the *Cgset4Δ* mutant (Fig. 2, *A* and *B*). In addition, while *CgSET4* expression was downregulated (Fig. 2*D*), *ScSET4* transcription was found to be activated upon azole exposure (33). These results highlight functional differences between the two proteins and are in accordance with other reported functions of ScSet4 protein. For example, ScSet4 plays an essential role in protection against oxidative stress by activating stress response genes (31), and 196 genes were found to be differentially regulated upon *ScSET4* disruption (34). Contrarily, *CgSET4* deletion had a minor effect on the *C. glabrata* transcriptome, with 48 genes displaying deregulation (Table S4). Moreover, *CgSET4* deletion did not lead to an increased susceptibility toward hydrogen peroxide–induced oxidative stress (Fig. 1*B*), which could in part be due to elevated expression of the *CgCTA1* gene in the *Cgset4Δ* mutant (Table S4). Furthermore, unlike *ScSET4* (31), ectopic expression of *CgSET4* was not found to be detrimental for cell growth in *C. glabrata* (Fig. 2*A*).

Despite these differences, Set4 in both *C. glabrata* and *S. cerevisiae* acts as a negative regulator of ergosterol biosynthesis (*ERG*) genes. *SET4* disruption led to azole resistance, with Set4 regulating *ERG3* and *ERG11* expression through direct binding to their promoters in *S. cerevisiae* (33). Ergosterol biosynthesis in fungi is a multistep energy-consuming process, with oxygen and heme acting as cofactors for many enzymes of the ergosterol synthesis pathway (Fig. S7) (50, 51). Furthermore, because of the centrality of ergosterol in maintaining fluidity, integrity, and functions of the plasma membrane, ergosterol biosynthesis is regulated at multiple levels, including transcriptional regulation, transport, and sterol feedback inhibition, and subcellular localization of enzymes (50, 51). The late steps of the ergosterol biosynthesis pathway involve many demethylation, reduction, and desaturation reactions (Fig. S7), with ergosterol being transported to the plasma membrane from the site of its synthesis, endoplasmic reticulum (50, 51).

**Figure S10:**
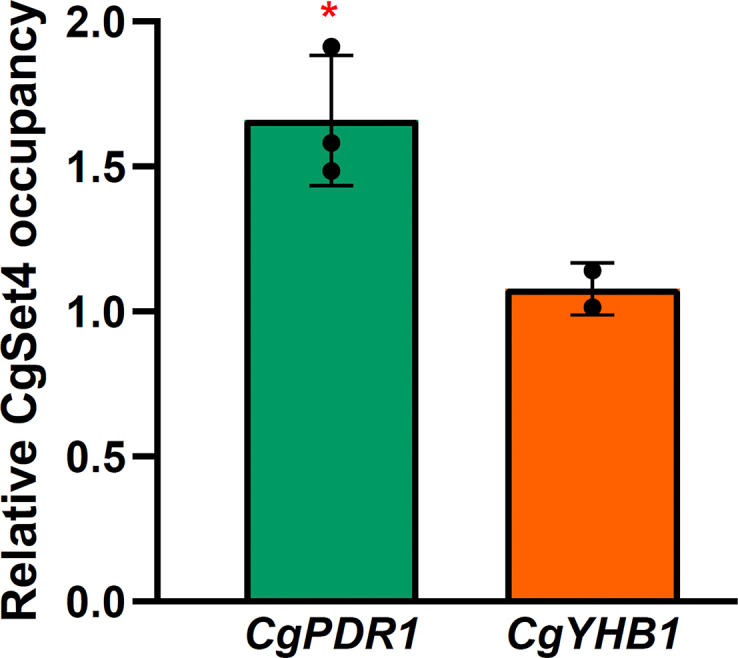
**ChIP analysis showing CgSet4-GFP occupancy on*****CgPDR1*****promoter.**

*ERG* gene expression is tightly regulated at the transcriptional level, with hypoxia and iron depletion resulting in downregulation of ergosterol synthesis in *S. cerevisiae* (31, 33, 49). Azole antifungals target a rate-limiting step of the ergosterol biosynthesis pathway that is mediated by the cytochrome P450–dependent lanosterol 14 alpha-demethylase enzyme, which is encoded by the *ERG11* gene (13, 14). Azole exposure in *C. glabrata* is known to result in elevated expression of *ERG* genes including *ERG11*, and this gene induction is largely carried out by the Zn2-Cys6 binuclear cluster transcription factor, CgUpc2a (21, 22, 23, 60). We show that *CgSET4* deletion led to the increased expression of *CgUPC2A*, along with its target *ERG* genes, *viz*., *CgERG2*, *CgERG3*, *CgERG4*, *CgERG6*, and *CgERG11* (Figs. 5*A* and 7*A*). In addition, elevated transcript levels of the zinc finger transcriptional activator gene *CgPDR1* as well as the multidrug transporter genes *CgCDR1* and *CgCDR2* (CgPdr1 target genes) in the *Cgset4Δ* mutant (Fig. 3*A*) suggest that CgSet4 is a general repressor of two major azole response pathways in *C. glabrata*. Consistent with this, ChIP analysis revealed CgSet4-GFP to be present at the promoter region of *CgPDR1* (Fig. S10). Of note, the CgSet1-dependent H3K4 methylation has recently been found to be increased on actively transcribing *ERG* genes in response to FLC (38), thereby highlighting the epigenetic regulation of *CgERG* genes in *C. glabrata*.

In *S. cerevisiae*, the major sterol regulator Upc2, a homodimer, is known to bind to the 7 bp sterol regulatory element (SRE) sequence TATACGA that is present in promoters of the *ERG*, sterol uptake, the DAN/TIR genes (50, 51). The C-terminal domain of Upc2 has been shown to act as an ergosterol-binding and sensing domain, with the ergosterol-bound Upc2 residing in the cytosol (50). Ergosterol depletion leads to the release of ergosterol, and the translocation of Upc2a to the nucleus, resulting in the transcriptional activation of its targets including *ERG* genes (50). The *C. glabrata* ortholog of *S. cerevisiae* Upc2, CgUpc2a, has recently been postulated to act like its *S. cerevisiae* counterpart and shown to regulate the expression of a vast array of genes including *ERG* and *CgPDR1* regulon genes, which contain SREs in their promoter regions (57).

Through ChIP-Seq analysis, Vu *et al.* (57) showed that CgUpc2a binds to about 1000 genes in *C. glabrata*. In addition, 64 genes including *CgERG4* were identified as indirect target genes of CgUpc2a (57). *CgUPC2A* itself was found to be upregulated upon FLC treatment, and its disruption rendered cells susceptible to both FLC and CSP drugs (57). In addition, although FLC-induced upregulation of *CgCDR1* and *CgPDR1* genes was lower and similar between *wt* and *Cgupc2aΔ* strains, respectively (57), a pivotal role for CgUpc2a in CgPdr1-dependent gene network was unveiled by the reduced binding of CgUpc2a to the mutated SRE in the *CgPDR1* promoter, as well as, by the diminished FLC-induced induction of the SRE-lacking *CgPDR1* gene (57). Our results of reduced *CgPDR1* gene expression in the double mutant *Cgset4Δupc2aΔ* (Fig. 7*D*) suggest that the elevated *CgUPC2A* transcript levels contribute to the increased *CgPDR1* gene expression in the *Cgset4Δ* mutant. Of note, further detailed investigations are required to determine if CSP susceptibility of the *Cgset4Δupc2aΔ* mutant is due to an imbalance of sterol species or cell wall components or both.

**Figure S11:**
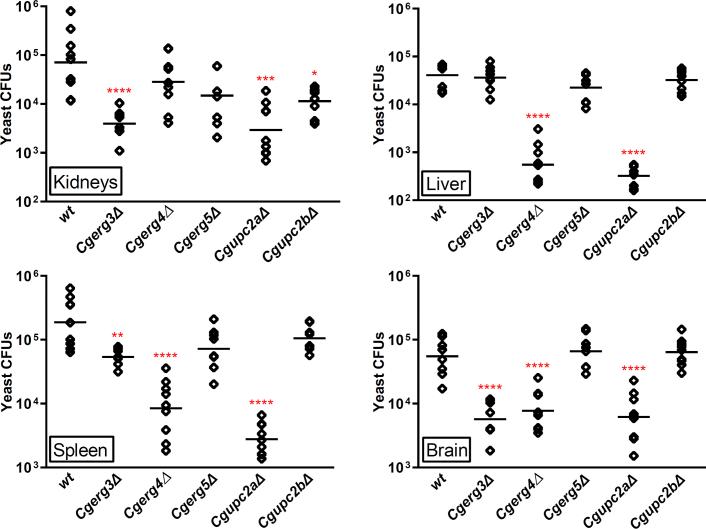
**Organ fungal burden in 6-8 week-old female BALB/c mice after 7 days of intravenous infection with indicated *C. glabrata* strains.**

In addition to the SET proteins, we have also investigated the role of three enzymes catalyzing late stages of ergosterol synthesis, CgErg3, CgErg5, and CgErg4, in antifungal tolerance and virulence. Intriguingly, while the *Cgerg3Δ* and *Cgerg4Δ* mutants exhibited elevated FLC sensitivity, the *Cgerg5Δ* mutant displayed FLC susceptibility, similar to that of the *wt* strain (Fig. 6, *A* and *C*). Furthermore, deletion of the *CgERG3*, *CgERG5*, and *CgERG4* genes led to decreased, increased, and increased susceptibility to CSP, respectively (Fig. 6*B*). Notably, the echinocandin-resistant isolates in a microevolution study, which exhibited crossresistance to FLC, have recently been shown to carry mutations in the *CgERG3* gene (53). Our mice infection studies revealed that in line with their distinct role in antifungal resistance, CgErg3 and CgErg4 were required for survival of *C. glabrata* in the murine model of systemic candidiasis in an organ-dependent manner, whereas CgErg5 was dispensable in this model (Fig. S11). In addition, whereas *CgUPC2A* deletion led to significantly attenuated survival of *C. glabrata* in various organs, *CgUPC2B* deletion affected survival adversely only in kidneys (Fig. S11), indicating that CgUpc2a plays a major role in virulence of *C. glabrata*. These results together also highlight the importance of ergosterol synthesis for *C. glabrata*–mammalian host interaction.

Furthermore, our data underscore that CgErg enzymes differ from one another in their requirement in cellular response to FLC and CSP. This difference among CgErg enzymes is likely to be determined by additional regulatory factors and/or other functions of CgErg proteins. These results are consistent with varied regulatory mechanisms of different *ERG* genes in *S. cerevisiae* and *C. glabrata* (21, 23, 33, 50, 57, 60). In this context, it is worth noting that despite CgUpc2a showing strong binding to the *CgERG1* gene promoter, FLC-induced activation of *CgERG1* gene was found to be similar between *wt* and *Cgupc2aΔ* mutant (57), thereby pointing toward the complex multifactorial environmental cue-dependent regulation of individual *CgERG* genes. Our data provide further support to this notion, as CgSet4 was found to be indispensable and dispensable for the CSP-induced repression of *CgERG1*, and other *CgERG* (*CgERG4*, *CgERG5*, *CgERG6*, *CgERG11*, and *CgERG25*) genes, respectively (Fig. 5, *C* and *D*).

CSP exposure has recently been reported to result in elevated reactive oxygen species production (61). Consistent with this, our transcriptional profiling analysis revealed upregulation of oxidative stress response genes in CSP-treated cells. Furthermore, a multifactorial role of mitochondria has recently been reported in CSP tolerance in *C. glabrata* (61). Given that a reduction in ergosterol content is known to adversely affect mitochondrial DNA maintenance (62), it is possible that elevated ergosterol in *Cgset4Δ* mutant modulates mitochondrial functions, which may contribute to CSP resistance in the mutant. Elucidation of the nexus among sterol metabolism, CSP resistance, and mitochondrial functions will shed light on the underlying molecular mechanism.

**Figure 8:**
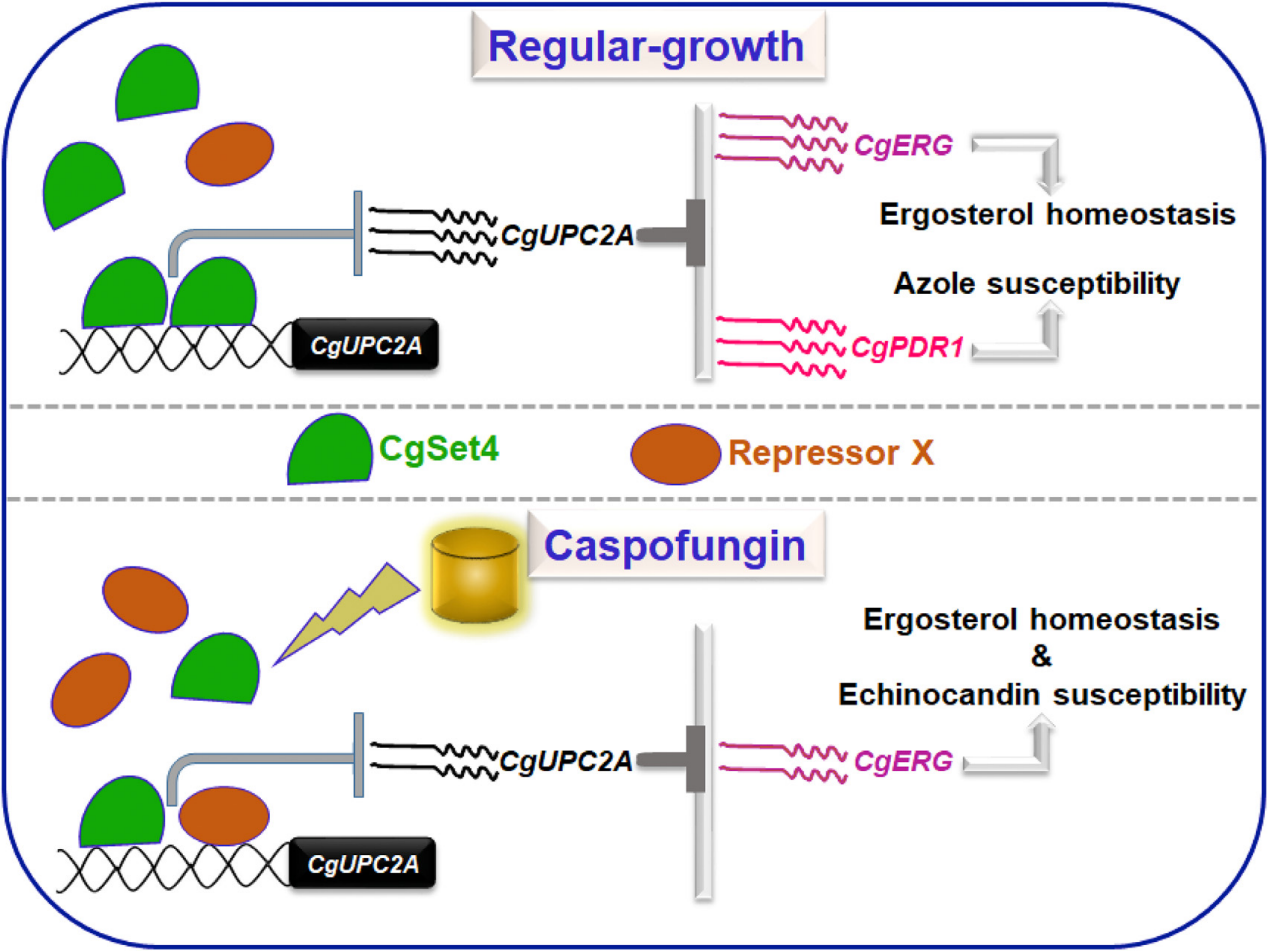
**A schematic summarizing key findings of the study.***Candida glabrata* cells maintain cellular ergosterol levels *via* tight regulation of ergosterol biosynthesis (*CgERG*) genes. CgSet4 acts as a repressor of CgPdr1-dependent multidrug resistance, and ergosterol biosynthesis pathways, as *CgSET4* deletion leads to increased basal expression of *CgPDR1* regulon and *CgERG* genes, elevated ergosterol content, and resistance to fluconazole and CSP antifungals. Under regular growth conditions, CgSet4 keeps *CgUPC2A* expression in check *via* binding to the *CgUPC2A* promoter. Since CgUpc2a is an activator of *CgERG* and *CgPDR1* genes, the lower levels of CgUpc2a result in restrained expression of *CgERG* and *CgPDR1* genes, thereby maintaining sterol homeostasis and susceptibility to azole antifungals. CSP exposure results in transcriptional and post-transcriptional downregulation of *CgSET4*, reduction in CgSet4 abundance on the *CgUPC2A* promoter, transcriptional activation and repression of *CgROX1* and *CgUPC2A*, respectively, and the consequent downregulation of *CgERG* genes. Two lines of evidence, the *Cgset4Δ* mutant’s proficiency in downregulating *CgERG* genes upon CSP exposure, and the decreased CgSet4 occupancy on *CgUPC2A* promoter in CSP-treated *wt* cells, point toward another repressor protein (repressor X) contributing to *CgUPC2A* and *CgERG* gene downregulation in response to CSP. This repressor is yet to be identified, although CgRox1, being an inhibitor of *CgERG* gene expression, appears to be a strong candidate for the same.

Collectively, our data suggest that the transcriptional regulation of *ERG* genes is not necessarily reflected in cellular ergosterol levels. Consistently, despite CgUpc2a regulating *ERG* gene expression, its disruption led to no significant decrease in ergosterol levels (44, 57). Similarly, cellular ergosterol levels were found to be decreased upon loss of both *UPC2* and *ECM22* (paralog of *UPC2*) genes, which code for activators of the sterol biosynthetic pathway in *S. cerevisiae* (50, 63). Based on our data, we propose that CgSet4 is a negative regulator of the basal-level expression of the *CgUPC2A* gene (Fig. 8), whereas the cellular response to CSP involves a wholesale downregulation of ergosterol biosynthesis pathway that is probably initiated by the transcriptional downregulation of *CgUPC2A* (Fig. 8). CSP-induced repression of *CgUPC2A* is probably primarily carried out by another repressor protein (Fig. 8), with CgRox1 to be a likely candidate whose expression was found to be upregulated in response to CSP exposure (Table S2). Of note, CgRox1 has recently been shown to be a negative regulator of *ERG* genes, with loss-of-function mutations in *CgROX1* rescuing the increased FLC susceptibility of the *Cgupc2aΔ* mutant (44). Furthermore, since the azole and CSP resistance of the *Cgset4Δ* mutant is reversed by *CgUPC2A* deletion (Fig. 7, *B* and *C*), CgUpc2a is likely to be the main effector protein of CgSet4, with CgUpc2a also controlling expression of the CgPdr1 regulon genes. Although the molecular basis for CgSet4-dependent regulation of CgPdr1 and CgUpc2a regulon genes is yet to be deciphered, our ChIP data suggest that it is likely to be through direct association of CgSet4 with the regulatory regions of *CgUPC2A*. However, the regulatory region of CgUpc2a, where CgSet4 could bind to, is yet to be identified.

Importantly, owing to a general repressive effect of CSP on *ERG* gene expression, our data underscore the need to revisit the combinatorial therapeutic regimen, which involves treatment of fungal infections with echinocandins along with other ergosterol-targeting drugs, polyenes (bind to ergosterol in the cell membrane), allylamines (inhibit squalene epoxidase, encoded by *ERG1*), or azoles (64, 65).

Altogether, our data demonstrate CgSet4 to be a key regulator of CgPdr1-dependent MDR and CgUpc2a-dependent ergosterol biosynthesis pathways, thereby making CgSet4 as a major component of the cell wall and cell membrane homeostasis systems in *C. glabrata*.

## Experimental procedures

### Strains, media, and growth

*C. glabrata* strains used in the study were derivatives of the BG2 strain and maintained in the rich YPD or minimal casamino acid (CAA) medium at 30 °C. The *Escherichia coli* DH5-α strain was used for plasmid propagation and maintained in LB medium at 37 °C. Overnight cultures of *C. glabrata* strains were grown for 3 to 4 h at 30 °C to obtain log-phase cultures. Antifungal drug and stress susceptibility was examined by serial dilution spotting assay and liquid medium–based growth analysis.

### *C. glabrata* gene deletion and cloning

The homologous recombination–based strategy was used to create *C. glabrata* single and double deletion strains using the *nat1* gene, which confers nourseothricin resistance, as a recyclable selection marker, as described previously (66). The *Cgset4Δ* strain was used as the parental strain to generate double mutants, *Cgset4Δcdr1Δ*, *Cgset4Δerg3Δ*, *Cgset4Δerg4Δ*, *Cgset4Δerg5Δ*, *Cgset4Δupc2aΔ*, and *Cgset4Δupc2bΔ*. For overexpression studies, *CgSET4* (*CAGL0G04499g*, 1.05 kb) gene was cloned under the strong *PDC1* promoter in the pRK1349 plasmid. For complementation studies, *CgSET4* gene was cloned with its own promoter (1 kb region upstream of the start codon ATG) in the pGRB2.1 plasmid at XbaI and XmaI restriction enzyme sites. The ergosterol biosynthesis genes *CgERG3* (*CAGL0F01793g*, 3.1 kb), *CgERG4* (*CAGL0A00429g*, 3.4 kb), *CgERG5* (*CAGL0M07656g*, 3.6 kb), *CgUPC2a* (*CAGL0C01199g*, 4.7 kb), and *CgUPC2b* (*CAGL0F07865g*, 4.5 kb) were cloned at XbaI–XmaI, XhoI–XmaI, SpeI–XmaI, XbaI–XmaI, and SpeI–XmaI restriction enzyme sites, respectively, in the pGRB2.2 plasmid. For generation of CgSet4-GFP construct, the *CgSET4* ORF (without the stop codon) was cloned downstream and upstream of the *PGK1* promoter and GFP-encoding region, respectively, at SpeI–XmaI restriction enzyme sites in the pGRB2.3 plasmid. The strains, plasmids, and primers used in this study are listed in Tables S8–S10, respectively.

### Protein extraction and immunoblotting

For protein expression studies, log-phase *C. glabrata* cells were collected and washed with PBS. Cells were lysed in the lysis buffer (20 mM Tris–HCl [pH 8.0], 100 mM NaCl, 1 mM EDTA, with 1 mM sodium orthovanadate, 1 mM PMSF, 10 mM sodium fluoride, and 1× protease inhibitor) mechanically using glass beads. After centrifugation of lysates for 15 min at 15,000 rpm at 4 ^ο^C, the supernatant was collected and resolved on SDS-PAGE. The proteins were transferred to the polyvinylidene difluoride membrane and probed with anti-H3K4me3 (Abcam; catalog no.: ab8580), anti-H3K36me3 (Abcam; catalog no.: ab9050), anti-histone H3 (Abcam; catalog no.: ab1791), anti-GFP (Abcam; catalog no.: ab290), or anti-Gapdh (Abcam; catalog no.: ab22555) antibodies.

### Mice infection assay

Mice infection studies were performed at the Animal House Facility of Centre for DNA Fingerprinting and Diagnostics (CDFD), Hyderabad, India in accordance with guidelines of the Committee for the Purpose of Control and Supervision of Experiments on Animals, Government of India, and were approved by the Institutional Animal Ethics Committee [EAF/RK/CDFD/15]. Briefly, *C. glabrata* strains were grown overnight in YPD or CAA medium, washed with PBS, and suspended in PBS. *C. glabrata* cells (4 × 10^7^; 100 μl PBS cell suspension) were injected into the tail vein of 6- to 8-week-old female BALB/c mice. Mice were monitored for 7 days, sacrificed, and four organs, kidneys, liver, brain, and spleen, were collected. Organs were homogenized in PBS, and appropriate dilutions were plated on penicillin- and streptomycin-containing YPD medium. Mouse organ fungal burden was determined by counting colonies manually.

### qRT–PCR

Total RNA was extracted from appropriate strains using the acid-phenol extraction method and digested with DNase I to eliminate any DNA contamination. DNase I-digested RNA (500 ng) was used to synthesize complementary DNA using the SuperScript III First-Strand Synthesis System for RT–PCR. The qPCR was performed using the SYBR green real-time PCR mastermix, and the sample C_T_ (cycle threshold) values were normalized against the C_T_ value of the control house-keeping *CgACT1* or *CgTDH3* gene. The fold change in expression under different conditions was determined using the comparative C_T_ (2^−ΔΔC^_T_) method.

### Microscopy analysis

The overnight grown *Cgset4Δ/CgSET4*-GFP strain was grown to log phase in CAA medium, followed by growth either in the absence (CAA) or the presence of FLC (64 μg/ml) or CSP (150 ng/ml), for 1 h. After incubation, cells corresponding to absorbance of 1.0 at 600 nm were collected, washed with PBS, and suspended in 100 μl of PBS containing 1 μg/ml Hoechst 33258 (Sigma; catalog no.: 94403) stain. After incubation at 37 °C for 15 min, 3 to 5 μl of stained cell suspension was mounted on a slide and imaged with the Confocal microscope (Zeiss LSM 700) equipped with 63×/1.44 numerical aperture objective.

### RNA-Seq analysis

Log-phase grown *wt* and *Cgset4Δ* mutant strains were grown with and without CSP (250 ng/ml) for 1 h. Total RNA was extracted using acid-phenol method followed by DNase digestion to remove any DNA contamination. The RNA samples were sent on dry ice to the AgriGenome Labs (a subsidiary of SciGenom Labs), Kakkanad, Kochi, Kerala, India (http://agrigenomelabs.com/), where these were further processed for sequencing. RNA quality was assured by taking RNA samples with RNA integrity number values ≥8. The complementary DNA library was prepared using TruSeq RNA Sample Prep Kits (Illumina), and 2 × 100 bp paired-end sequencing was performed on the Hiseq 2500 Illumina platform. The 40 to 60 million high-quality reads were obtained for each sample. The sequences were trimmed to remove unwanted sequences and aligned pairwise with the *C. glabrata* CBS138 reference genome (http://www.candidagenome.org). The DESeq analysis package was used, and genes that exhibited at ≥1.5-fold change in expression (*q* value = ≤0.05) were considered as DEGs.

### Ergosterol estimation

The ergosterol content in *C. glabrata* strains was measured, as described previously (67). Briefly, strains were grown to the log phase in the CAA medium (150 ml) at 30 °C, and absorbance at 600 nm of cultures was measured. After an absorbance at 600 nm normalization, cells were pelleted down and the wet weight of the cell pellet was determined. The cell pellets were suspended in freshly prepared alcoholic potassium hydroxide in sterile borosilicate glass screw-cap tubes and incubated at 85 °C in a water bath for 3 h. After cooling to room temperature, the water and *n*-heptane (1:3 ratio) mixture was added to each tube, and tubes were vortexed vigorously for 3 min. The samples were kept static for about 15 min for phase separation, and the upper transparent layer of *n*-heptane was collected. The UV spectrophotometric profiles were recorded between 220 and 300 nm, with both ergosterol and 24 (28)-dehydroergosterol (DHE) absorbing at 281.5 nm, whereas 24(28)-DHE alone absorbing at 230 nm. The ergosterol content was determined by subtracting the amount of 24 (28)-DHE (calculated from the absorbance at 230 nm) from the total ergosterol plus 24 (28)-DHE content (calculated from the absorbance at 281.5 nm), and calculated as a percentage of the wet weight of the cells using the following equation:

[{(A281.5/290) × F}/pellet weight] − [{(A230/518) × F}/pellet weight], where F is the factor for dilution in petroleum ether, whereas 290 and 518 are the E values (in percent per centimeters) determined for crystalline ergosterol and 24(28)-DHE, respectively.

### ChIP assay

Log-phase *C. glabrata* strains were grown in the presence or the absence of CSP (250 ng/μl) for 1 h. Cells were crosslinked with 1% formaldehyde for 20 min, followed by quenching with 125 mM glycine for 10 min. Cell pellets were collected and lysed in FA lysis buffer (1 mM EDTA [pH 8.0], 50 mM Hepes [pH 7.5], 140 mM NaCl, 0.1% sodium deoxycholate [w/v], 1× protease inhibitor, and 1% Triton X-100) by bead-beating. After removal of the cell debris by centrifugation, the supernatant was subjected to sonication for 40 min, with 30 s pulses of on and off at highest amplitude. The samples were centrifuged at 15,000 rpm for 10 min, and the soluble fraction was collected. The 1/10th volume of the soluble fraction was saved as “input” fraction, and the remaining soluble fraction was precleared with protein-A Sepharose beads, prior to immunoprecipitation with anti-GFP antibody for 4 h at 4 °C. After incubation, beads were given four consecutive washes, with FA lysis buffer, lysis buffer containing 0.5 M NaCl, wash buffer (100 mM Tris–HCl [pH 8.0], 0.25 M LiCl, 0.5% NP-40, 0.5% deoxycholic acid [w/v], and 1 mM EDTA) and TE buffer. The beads were suspended in the elution buffer (50 mM Tris–HCl, 10 mM EDTA, and 1% SDS) and incubated overnight at 65 °C for decrosslinking, followed by proteinase K treatment for 1 h. DNA was precipitated using phenol:chloroform:isoamyl alcohol, suspended in TE, treated with RNAse at 37 °C for 1 h, and used as template for qRT–PCR using appropriate set of primers.

### Other procedures

The cell wall analysis was performed, as described previously (66).

### Statistical and functional analysis

The statistical significance was determined using the GraphPad Prism software (GraphPad Software, Inc). The two-tailed Student’s *t* test and the non-parametric Mann–Whitney test were used for intergroup comparisons and mouse organ fungal burden analysis, respectively. DEGs were analyzed and functionally annotated using the Candida Genome Database (http://www.candidagenome.org) and DAVID (https://david.ncifcrf.gov/) tools.

## Data availability

The raw RNA-Seq data have been submitted to the National Center for Biotechnology Information Gene Expression Omnibus repository (https://www.ncbi.nlm.nih.gov/geo/), with Gene Expression Omnibus accession number GSE202654.

## Supporting information

This article contains supporting information (27, 50, 68, 69, 70, 71) .

## Conflict of interest

The authors declare that they have no conflicts of interest with the contents of this article.
