# Supplementary material for: The SET-domain protein CgSet4 negatively regulates antifungal drug resistance via the ergosterol biosynthesis transcriptional regulator CgUpc2a
Source: J Biol Chem. 2022 Sep 13;298(10):102485. doi: 10.1016/j.jbc.2022.102485 (PMC9576903; doi:10.1016/j.jbc.2022.102485)
Supplement: Supporting information [file mmc1.docx]

**The SET-domain protein CgSet4 negatively regulates antifungal drug resistance via the ergosterol biosynthesis transcriptional regulator CgUpc2a**

Priyanka Bhakt^1,2^, Mayur Raney^1,3^, Rupinder Kaur^1,*^

^1^Laboratory of Fungal Pathogenesis, Centre for DNA Fingerprinting and Diagnostics, Hyderabad-500039, India

^2^Present address: Department of Biology, Drexel University, Philadelphia, PA-19104, USA

^3^Graduate studies, Manipal Academy of Higher Education, Manipal-576104, Karnataka, India

^*^For correspondence: Rupinder Kaur, Centre for DNA Fingerprinting and Diagnostics (CDFD), Hyderabad, Telangana, India. Tel.: 91-40-27216137; Fax: 91-40-27216006; E-mail: [rkaur@cdfd.org.in](mailto:rkaur@cdfd.org.in).

**Supporting Information**

**Supporting figures: S1-S11**

**Supporting tables: S1-S10**

**Supporting figure legends**

**Figure S1: *C. glabrata* contains six SET domain-containing proteins.**

1. Multiple amino acid sequence alignment of the SET domain of CgSet1-CgSet6 proteins, as determined by the Clustal Omega program. The first and the second boxed regions mark the conserved amino acids involved in S-adenosylmethionine (SAM) and lysine binding, respectively, in the canonical SET domains.
2. Schematic depiction of the domain structure of SET domain-containing proteins, as predicted by the SMART tool (http://smart.embl-heidelberg.de). Amino acid positions of predicted domains are indicated. The orange-colored bar represents low complexity region. Images are not drawn to scale.

**Figure S2: CgSet4 negatively regulates fluconazole and caspofungin resistance in clinical strains of *C. glabrata.***

1. Serial dilution spotting analysis illustrating fluconazole susceptibility of indicated *C. glabrata* strains. Fluconazole was used at a concentration of 16 µg/ml (FLC-16), 32 µg/ml (FLC-32), and 64 µg/ml (FLC-64) in YPD medium. Images were captured after growth was recorded after 1-2 days of growth at 30^o^C. CBS138, YRK2289 and YRK2291 strains have been isolated from human feces, vitreous fluid and corneal scraping, respectively. *CgSET4* was expressed from *PDC1* promoter in all strains.
2. Liquid medium-based growth analysis illustrating caspofungin susceptibility of indicated *C. glabrata* strains. Strains were cultured at 30^ο^C in CAA medium lacking (CAA) or containing 75 ng/ml (CSP-75) or 150 ng/ml (CSP-150) caspofungin for 16 h. After incubation, cultures were diluted in PBS, and 3 µl of undiluted, and 5-, 25-, 125-and 625-fold diluted cultures were spotted on CAA medium, and growth was recorded after 1 day of growth at 30^o^C.

**Figure S3: CgSet4-GFP complements decreased fluconazole susceptibility of the *Cgset4Δ* mutant.**

1. Serial dilution spotting analysis illustrating fluconazole susceptibility of indicated *C. glabrata* strains. Fluconazole was used at a concentration of 16 µg/ml (FLC-16), 32 µg/ml (FLC-32), and 64 µg/ml (FLC-64) in CAA medium.
2. Immunoblot analysis showing enrichment of CgSet4-GFP in the chromatin fraction of *wild-type* (*wt*) cells expressing CgSet4-GFP. Logarithmic-phase cells were spheroplasted with zymolyase (2 µg/µl) at 37^ο^C for 1 h, and suspended in lysis buffer (0.5 mM spermidine, 1 mM β-mercaptoethnol, 0.1% NP 40, 50 mM NaCl, 10 mM Tris-HCl pH 8.0, 5 mM MgCl2, 5 mM CaCl2, and freshly added 1x protease inhibitor mix). After incubation at 4^ο^C for 2 h, the supernatant was collected as the Whole cell lysate fraction. This lysate was centrifuged at 14,000 rpm for 5 min at 4^ο^C, followed by collection of the supernatant (Soluble fraction), and suspension of the pellet in lysis buffer (Pellet fraction; represents chromatin fraction). 15 µl of each fraction was loaded on 12% of SDS-PAGE and probed with indicated antibodies. Histone H3 was used as a positive control for fractionation. CgSet4-GFP, histone H3 and GAPDH proteins correspond to 65, 15 and 36 kDa bands, respectively.

**Figure S4: Flow cytometry-based measurement of cell wall components.** CAA-medium grown log phase cells of *wt* and *Cgset4Δ* strains were collected and stained with 2.5 μg/ml calcofluor white (for chitin estimation), 12.5 mg/ml aniline blue (for β-glucan estimation) and 4 μg/ml concanavalin A (for mannan estimation). Data (mean ± SEM, n = 4) are presented as the mean fluorescence intensity ratio, which was determined by dividing the fluorescence intensity value of the mutant sample by that of the *wt* sample (considered as 1.0). **, p<0.01; paired two-tailed Student's t-test.

**Figure S5: Colony-forming unit assay-based viability analysis.** CAA-medium grown log phase cells of *wt* and *Cgset4Δ* strains were grown either in the absence (CAA) or presence of 250 ng/ml caspofungin (CSP) for indicated time intervals. At each time, a culture aliquot was taken out, diluted in PBS and appropriate dilution was plated on the YPD medium. After 24 h growth at 30^ο^C, colonies were counted, and the number of viable cells was calculated by multiplying the colony number by the appropriate dilution factor. Data (mean ± SD; n =2;) represent colony forming units/ml of the culture.

**Figure S6: Heat map illustrating expression of genes of the ergosterol biosynthesis pathway in indicated strains, as determined by RNA-Seq.** The raw FPKM values were Z-score transformed.

**Figure S7: A schematic representation of the ergosterol biosynthesis pathway in yeast**. The ergosterol synthesis pathway consists of three modules, which are typified by the site of synthesis and the product formed, and are represented by three boxes. The first part (blue box) involves mevalonate synthesis which occurs mostly in the mitochondria. The second section (pale yellow box) is dedicated to farnesyl pyrophosphate synthesis which occurs in the vacuole or the cytoplasm. The late pathway (green box) involving ergosterol synthesis is the last module, and is mainly carried out in the Endoplasmic reticulum. Many biomolecules including dolichol and ubiquinone are formed from farnesyl pyrophosphate. The enzymes along with their requirement for oxygen, heme and iron to catalyze biosynthesis reactions are marked. This figure is adapted from Jordá and Puig, Genes, 2020 (50).

**Figure S8: Liquid medium-based growth analysis illustrating caspofungin susceptibility of the *Cgupc2aΔ* mutant.** *wt* and *Cgupc2aΔ* strains were cultured at 30^ο^C in CAA medium lacking (CAA) or containing 75 ng/ml (CSP-75), 125 ng/ml (CSP-125) or 150 ng/ml (CSP-150) caspofungin for 16 h. After incubation, cultures were diluted in PBS, and 3 µl of undiluted, and 10-, 100-, 300- and 500-fold diluted cultures were spotted on CAA medium, and growth was recorded after 1 day of growth at 30^o^C.

**Figure S9: Serial dilution spotting analysis illustrating attenuated growth of the *Cgset4Δupc2aΔ* mutant in YPD medium**. Indicated *C. glabrata* strains were grown overnight in YPD medium, and cultures were normalized to an OD_600_ of 1.0. Cultures were 10-fold serially diluted, and 3 µl of each dilution was spotted on YPD medium. Images were captured after 1 days of growth at 30^o^C.

**Figure S10:** ChIP analysis showing CgSet4-GFP occupancy on *CgPDR1* promoter (5'UTR region) in log-phase YPD medium-grown cells of the *Cgset4Δ*/*CgSET4-GFP* strain using anti-GFP antibody. The *Cgset4Δ*/Vector was used as control. The percentage of input was calculated for each IP and the ChIP amplification were normalized to the DNA input samples. Data (mean ± SD; n = 2-3) represent CgSet4 occupancy in *Cgset4Δ*/*CgSET4-GFP* strain, compared to *Cgset4Δ*/Vector samples. The primers used detected the promoter regions of *CgPDR1* and *CgYHB1* genes. *CgYHB1* gene promoter-specific primers were used as negative control. *, p<0.05, paired two-tailed Student's t-test.

**Figure S11: Organ fungal burden in 6-8 week-old female BALB/c mice after 7 days of intravenous infection with indicated *C. glabrata* strains (4x10^7^ cells**). Diamonds and bars denote CFUs recovered from target organs of the individual mouse, and the CFU geometric mean (n = 7-10), respectively, for each organ. *, p<0.05; **, p<0.01; ***, p<0.001; ****, p<0.0001, Mann-Whitney test.

**Supporting tables: S1-S10**

**Table S1:** A list of six *C. glabrata* SET domain-containing proteins.

**Table S2:** A list of differentially expressed genes (DEGs) in the *wild-type* strain in response to caspofungin exposure.

**Table S3:** Enriched GO terms for biological process (BP), cellular component (CC) and molecular function (MF) categories for proteins, as determined by the DAVID tool, encoded by genes that are upregulated (**A**) and downregulated (**B**) in response to caspofungin exposure in *wild-type* cells.

**Table S4:** A list of differentially expressed genes (DEGs) upon *CgSET4* deletion.

**Table S5:** Enriched GO terms for BP, CC and MF categories for proteins, as determined by the DAVID tool, encoded by genes that are upregulated (**A**) and downregulated (**B**) upon *CgSET4* deletion.

**Table S6:** A list of differentially expressed genes (DEGs) in the *Cgset4Δ* mutant in response to caspofungin exposure.

**Table S7:** Enriched GO terms for BP, CC and MF categories for proteins, as determined by the DAVID tool, encoded by genes that are upregulated (**A**) and downregulated (**B**) in the *Cgset4Δ* mutant in response to caspofungin exposure.

**Table S8:** A list of strains used in the study.

**Table S9:** A list of plasmids used in the study.

**Table S10:** A list of primers used in the study.
